# Fluoroquinolones Hybrid Molecules as Promising Antibacterial Agents in the Fight against Antibacterial Resistance

**DOI:** 10.3390/pharmaceutics14081749

**Published:** 2022-08-22

**Authors:** Ioana-Andreea Lungu, Octavia-Laura Moldovan, Victoria Biriș, Aura Rusu

**Affiliations:** 1 The Doctoral School of Medicine and Pharmacy, George Emil Palade University of Medicine, Pharmacy, Science, and Technology of Targu Mures, 540142 Targu Mures, Romania; 2 Discipline of Pharmaceutical and Therapeutical Chemistry, Department F2, George Emil Palade University of Medicine, Pharmacy, Science, and Technology of Targu Mures, 540142 Targu Mures, Romania

**Keywords:** hybrids, antibiotic hybrids, fluoroquinolones, fluoroquinolones hybrids, antibacterial agents, antibacterial resistance, structure–activity relationship

## Abstract

The emergence of bacterial resistance has motivated researchers to discover new antibacterial agents. Nowadays, fluoroquinolones keep their status as one of the essential classes of antibacterial agents. The new generations of fluoroquinolones are valuable therapeutic tools with a spectrum of activity, including Gram-positive, Gram-negative, and atypical bacteria. This review article surveys the design of fluoroquinolone hybrids with other antibacterial agents or active compounds and underlines the new hybrids’ antibacterial properties. Antibiotic fluoroquinolone hybrids have several advantages over combined antibiotic therapy. Thus, some challenges related to joining two different molecules are under study. Structurally, the obtained hybrids may contain a cleavable or non-cleavable linker, an essential element for their pharmacokinetic properties and mechanism of action. The design of hybrids seems to provide promising antibacterial agents helpful in the fight against more virulent and resistant strains. These hybrid structures have proven superior antibacterial activity and less susceptibility to bacterial resistance than the component molecules. In addition, fluoroquinolone hybrids have demonstrated other biological effects such as anti-HIV, antifungal, antiplasmodic/antimalarial, and antitumor activity. Many fluoroquinolone hybrids are in various phases of clinical trials, raising hopes that new antibacterial agents will be approved shortly.

## 1. Introduction

Although the discovery of antibiotics revolutionized medicine, even nowadays, the threat of bacterial infections is by no means an insignificant one. Healthcare-associated infections represent just a segment of a substantial burden worldwide, each year affecting hundreds of millions of patients worldwide [1]. Each year, surgical site infections threaten the lives of millions of patients and contribute to the development of antimicrobial resistance [2]. To combat infectious diseases, the need for efficient antimicrobial therapies is compelling.

The antibacterial quinolones (QNs) and further developed fluoroquinolones (FQNs) represent one of the most important classes of antimicrobial agents from many points of view: activity spectrum, administrations, and tissue distribution, being primarily used to fight bacterial infections. Moreover, their versatile molecules allowed improvements in both the pharmacokinetic and pharmacodynamic properties. Nowadays, FQNs are actively prescribed to treat various diseases caused by Gram-negative and Gram-positive bacteria, such as urinary infections, respiratory tract infections, and several gastro-intestinal tract infections [3]. Unfortunately, similar to other antibiotics, a few concerns do not spare FQN usage. Widespread use in humans and animals has determined the appearance of antibacterial resistance toward FQNs [4,5].

The need for the continuous discovery of new derivatives has arisen, having considered this major threat, to keep up with the adaptation of bacteria. Certain FQNs have potential therapeutic uses, addressing a wide range of pathologies, such as bacterial infections, tuberculosis, malaria, viral infections (e.g., hepatitis, HIV, herpes), fungal infections, cancer, immunodepression, and neurodegenerative diseases. Moreover, the chemical properties of FQNs, particularly their reactivity and structure, have also sparked interest, maintaining longstanding attention towards this class for several decades [6].

Hybrids represent a particular class that could be obtained using FQNs as one of the antibiotic components. A hybrid antibiotic can be defined as two or more molecules or pharmacophores linked together, synthesized to exhibit a desired antimicrobial effect. Considering that the emergence of antibacterial resistance is better suppressed by combination therapy rather than monotherapy, it is hypothesized that through hybridization, additional benefits that were missing in individual molecules are obtained [7].

This review aims to present hybridization design strategies based on QN and FQN derivatives used in the development of antibacterial agents and highlight the hybrids’ biological effects, emphasizing the antibacterial effect. In addition, this paper highlights the need for new antimicrobial drugs and the potential that hybridization has as a strategy in the context of antimicrobial resistance as a global phenomenon.

## 2. The Research Methodology and Literature Review

This review is based on relevant articles from the following databases: Clarivate Analytics Web of Science, PubMed, Elsevier, Scopus, and ScienceDirect. The selected papers concerning hybrids were mainly published over the last two decades. In addition, relevant publications regarding the topics of “fluoroquinolones” and “antibiotic resistance”, “antimicrobial resistance”, and “antibacterial resistance” were taken into consideration. The search methodology used in the first stage used the following keywords: “hybrid”, “fluoroquinolones”, and “antibacterial agents”. Then, other keywords related to the results of a primary search (mainly primary units of the identified hybrids) were used.

The publications were selected if they included relevant data regarding the aspects referred to in our review: synthesis of hybrids comprising QNs/FQNs and other structures and biological activity evaluation of the obtained compound (mainly focused on the antibacterial activity).

The chemical structures were sketched with Biovia Draw (https://discover.3ds.com/biovia-draw-academic (accessed on 27 June 2021)).

## 3. Antibacterial Quinolones (QNs)

From the discovery of nalidixic acid until the synthesis of the newest FQNs, these synthetic antibacterial agents have proved to be a valuable tool in the fight against infections [8,9,10,11,12]. Following Lesher’s discovery of nalidixic acid in 1962 as an antibacterial agent, the discovery of 6-fluoro analogues has given rise to the FQNs class, one of the most commonly used antibiotic classes [8,13,14,15,16,17]. Thus, fluorinated compounds opened the road to further generations, improved pharmacokinetic and pharmacodynamic profiles, and provided a broader antibacterial spectrum. As a result, the class is generically called “fluoroquinolones” because all representatives are mostly fluorinated structures [9,18].

Third-generation representatives (e.g., levofloxacin) are active against *streptococci* [19]. Noting that the introduction of a fluorine atom increased the efficiency of flumequine (first generation), the following optimized compounds had a fluorine atom at the C6 position. More efficient FQNs of the second generation (norfloxacin, ciprofloxacin, and ofloxacin) and third generation (levofloxacin) were obtained. Exceptionally, temafloxacin (second generation) presented three fluorine atoms in its chemical structure. Unfortunately, temafloxacin was withdrawn due to cardiotoxicity. Additionally, the producer withdrew clinafloxacin (a third-generation chlorofluoroquinolone) due to the adverse effects of phototoxicity and hypoglycemia [20,21]. Fourth-generation FQNs can have more than one fluorine atom or chlorine atom [22,23]. The advantages of FQNs are essential: slower development of bacterial resistance given the action on DNA gyrase and Topoisomerase IV, and activity against anaerobic bacteria in addition to Gram-negative and Gram-positive strains [19,24]. Over the last two decades, representatives such as besifloxacin, finafloxacin, delafloxacin, and zabofloxacin have received approval for therapy. Presently, the classification by the generation of these new compounds is controversial. They are often reported as belonging to the fourth generation and less frequently to a new generation (the fifth). Even though the mechanism of action for these new compounds does not bring essential new elements, there is the question of shaping the fifth generation, considering the broad spectrum of activity (including resistant bacterial strains) and higher potency. Moreover, these new representatives present a low risk for bacterial resistance development [25].

### 3.1. Structural Characterization of Antibacterial QNs

**Figure 1:**
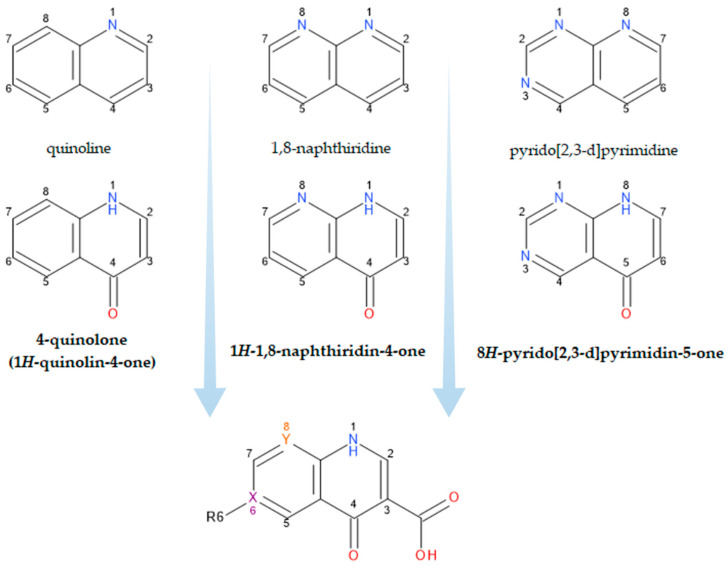
Structural precursors and general structure of QNs (FQNs if R6 = F); X, Y = C or N.

What we now generically term “quinolones” are, in fact, derivatives of either 4-quinolone, 1,8-naphthyridine-4-one, or pyrido-pyrimidine-5-one structures (Figure 1) [19,26].

The classification according to the chemical structure of the basic nucleus (Figure 1) includes the following groups of compounds:

- Naphthyridine derivatives (nalidixic acid, enoxacin, trovafloxacin, zabofloxacin);
- Quinoline derivatives (cinoxacin);
- Pyrido-pyrimidine derivatives (pyromidic acid, pipemidic acid);
- Quinoline derivatives (norfloxacin, ciprofloxacin, enrofloxacin, moxifloxacin, besifloxacin, delafloxacin, finafloxacin, lascufloxacin, nemonoxacin);
- Compounds with different structures (flumequine, ofloxacin, marbofloxacin, nadifloxacin, and levonadifloxacin).

A contradiction between the generic name “quinolones” and the exact name of the compounds belonging to this class is observed [8,20,25,27,28,29].

An attempt at classification by the number of fluorine atoms in the chemical structure of FQNs includes:

- Non-fluorinated quinolones (nemonoxacin);
- Monofluoroquinolones (ciprofloxacin, enoxacin, marbofloxacin, moxifloxacin, finafloxacin, pradofloxacin, nadifloxacin and levonadifloxacin, zabofloxacin);
- Difluoroquinolones (lomefloxacin, sarafloxacin, sparfloxacin, garenoxacin);
- Trifluoroquinolones (fleroxacin, temafloxacin, trovafloxacin, lascufloxacin);
- Monochloro- and monofluoroquinolones (besifloxacin);
- Monochloro- and difluoroquinolones (sitafloxacin);
- Monochloro- and trifluoroquinolones (delafloxacin) [25,28,29].

The chemical structure of this class of compounds is based on the 1,4-dihydro-pyridine-4-one nucleus, essential for antibacterial activity. The biological activity of a QN is determined by the following important structural elements: (a) the pyridinic ring, unsaturated between the C2 and C3 positions, the presence of a 4-oxo functional group, substitution at the N1 position; and (b) an aromatic B ring. Positions C2, C3, and C4 determine the antibacterial activity (influences the affinity towards bacterial enzymes) [18,27]. In addition, positions C3 and C4 are involved in metal chelation and other interactions with di- and trivalent cations [30]. The newest FQNs’ structural characteristics are described in detail by Rusu A. et al. (2021) [25].

**Figure 2:**
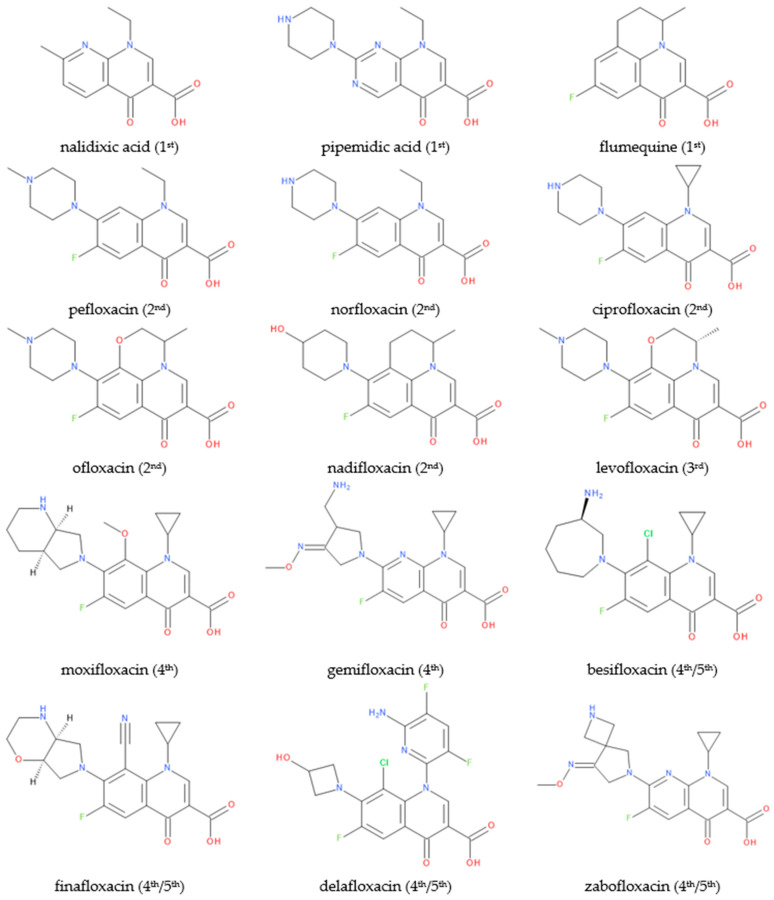
Chemical structures of (F)QNs; the generation is mentioned in parentheses.

FQN derivatives are amphoteric compounds whose chemical structure has a carboxyl group at the C3 position (essential for antibacterial activity on the DNA gyrase target). Most commonly, FQNs contain a heterocycle with nitrogen at the C7 position [29]. However, various other radicals have been linked to the general structure over time; the chemical structures of relevant representatives are presented in Figure 2.

There are essential structural elements (C2, C3, and C4 positions) of FQNs closely related to the mechanism of action. In addition, the C1, C5, C7, and C8 positions can serve as targets for various potential substituents (Table 1) [9].

Over time, researchers have explored numerous possibilities for modifying the basic structure of QNs. Researchers have tested various substitutions to obtain a molecule with potent antibacterial activity, a broad spectrum of activity, and superior pharmacokinetic properties. Numerous new compounds have been synthesized and tested for their biological activity, with the ultimate goal being the perfect “X-floxacin”.

### 3.2. Physicochemical Properties of FQNs

The antibacterial QNs class contains crystalline substances or crystalline powders, generally white or yellowish-white in color, tasteless, with a slightly bitter or bitter taste, odorless, insoluble in water and slightly soluble in common organic solvents, and soluble in dimethyl sulfoxide. Their solubility increases in acidic and basic environments (QNs form water-soluble salts). Many QNs are conditioned as salts: hydrochlorides (ciprofloxacin, moxifloxacin, besifloxacin, lascufloxacin), malate hemihydrates (nemonoxacin), methanesulfonates (pefloxacin), and toluenesulfonates (tosufloxacin) [25,36,37,38,39,40].

The low water solubility of FQNs (except their salts) is due to the crystalline structure with condensed aromatic nuclei. FQNs present high melting points (greater than 200 °C) due to a stable crystalline structure. Their water solubility depends on pH (as amphoteric compounds): in acidic or basic environments, they dissolve, forming salts; in the range of pH 6–8, water solubility is low [18,28]. Increasing the solubility of FQNs is very important for their parenteral administration. For this purpose, new strategies were developed, such as obtaining prodrug formulations [41,42,43].

Some FQNs form hydrates depending on the temperature and relative humidity. Lambert A. et al. (2007) confirmed the predominance of the zwitterion form of levofloxacin in water and its lipophilic character, providing models of hydrated molecules with five water molecules [44].

FQNs present one or two chiral centers in the chemical structure and are available as racemates (ofloxacin, gemifloxacin, nadifloxacin), enantiomers (levofloxacin, moxifloxacin), or diastereoisomers (besifloxacin) [18,25,45]. It is known that spatial conformation influences the physical properties of molecules. Thus, an increase in the water solubility of the enantiomers was noted, with the racemates having reduced solubility (e.g., levofloxacin versus ofloxacin) [46,47].

FQNs derivatives are amphoteric compounds with four different chemical species in solution (cationic, anionic, zwitterionic, and neutral). These distinct molecular species have different properties in terms of solubility and lipophilicity. The ionized forms are much more soluble in water, and the neutral forms are more lipophilic. Two pKa values are most frequently reported. A value is conferred by the three-carboxyl group, which gives the molecule an acidic character. The nitrogen atoms confer another value from the heterocyclic substituents in C7 position (piperazine, pyrrolidine, etc.). Rusu et al. (2011) established three protonation centers by 1H NMR-pH titrations (the carboxylate moiety, the N-1′ and N′-4-piperazine nitrogens) for six FQNs. Additionally, macro- and microprotonation schemes and species-specific diagrams have been outlined [48,49]. Knowledge of the intimate protonation processes of FQNs is crucial in facilitating diffusion through membranes under particular conditions, increasing the distribution and accumulation in different target tissues, binding to structural components of membranes or specific intracellular ligands, and interpreting chemical structure–biological activity relationships [48,50].

The fluorine atom is often identified in the lead optimization studies as a strategy to increase the lipophilicity (log P) of the compound, block the metabolism, or optimize the pharmacokinetic properties [51,52,53,54]. The introduction of the fluorine atom at the C6 position led to an increased antimicrobial activity versus non-fluorinated QNs; it increased the degree of penetration into the bacterial cell and the activity against Gram-negative bacteria [55]. Several FQNs from the second generation were found to be lipophilic compounds (e.g., pefloxacin), intermediate lipophilic compounds (e.g., ciprofloxacin and ofloxacin), and hydrophilic compounds (e.g., norfloxacin) based on the true partition coefficients [56].

The physicochemical parameters of lomefloxacin, levofloxacin, and moxifloxacin, as potential bioavailability descriptors, were determined (in vitro and in silico) in a study performed by Kłosińska-Szmurło, E. et al. (2014) [57]. These published data concerning the lipophilic character are in agreement with the following order enrofloxacin > levofloxacin > ciprofloxacin > norfloxacin, established later by Blokhina S.V. et al. (2016) [47]. Some new FQNs are more lipophilic than others based on the experimental or calculated log P values. An ordering of the new compounds according to their lipophilic character is as follows: nadifloxacin > lascufloxacin > delafloxacin > besifloxacin > nemonoxacin > finafloxacin > zabofloxacin [25]. Currently, numerous structural modifications of FQNs are being studied to increase the lipophilicity of the molecule (e.g., new derivatives, FQNs hybrids, prodrugs) [58].

Another characteristic property of FQNs is the ability to be complexed by metal ions. Due to the carboxylic group at the C3 position, the piperazinyl ring (or another N-heterocycle) at the C7 position, and the carbonyl oxygen atom at the C4 position, FQNs could form metal complexes. FQNs act as bidentate, unidentate, or bridging ligand. The stoichiometry of the chelated forms depends on several factors: the relative concentrations of the chelating agents (FQNs), the metal ions, the valence of the metal ion, and the pH value. FQNs can form 1:1, 2:1, or 3:1 chelates with metal ions. Over time, research on metal ion complexation has focused on numerous chemical elements [30,38,59].

### 3.3. Mechanism of Action of Antibacterial FQNs

Antibacterial FQNs have a bactericidal effect involving a particular mechanism of action, namely the inhibition of DNA replication and transcription [30,60]. This mechanism is carried out by interacting with complexes of DNA and the enzymes DNA gyrase (a type II Topoisomerase) and Topoisomerase IV. These are two essential enzymes involved in DNA cleavage and ligation reactions [24,32,61]. The two enzymes have a similar tetrameric structure (A2B2). Gyrase’s subunits are GyrA and GyrB while Topoisomerase IV’s subunits are ParC and ParE. The GyrA subunit of gyrase contains the active site with tyrosine residuals. In contrast, the subunit GyrB contains the TOPRIM domain where the divalent ions bind, making the processes of DNA cleavage and ligation possible. The ParC subunits of Topoisomerase IV are responsible for DNA binding and the cleavage and re-ligation reaction. The ParE subunits are responsible for ATP binding and hydrolysis. These two enzymes ensure the double helix passes through a temporary double-stranded break to make DNA replication possible. The genomic integrity through this process is maintained by connecting the enzymes (the active site contains tyrosine residues) to the DNA strands through covalent bonds, forming complexes known as “cleavage complexes” [30,61,62].

Although similar in structure and mechanics, the two enzymes’ particular function in DNA replication differs [32,63,64,65]. DNA gyrase is a unique enzyme in bacterial cells but not in the higher eukaryotes. It is the only Topoisomerase that can introduce negative supercoils into DNA using energy from ATP hydrolysis. It is primarily responsible for releasing the tension that accumulates in front of the replication forks [5,32,61,66]. Topoisomerase IV plays a role in relaxing positive supercoils in the DNA. This enzyme removes knots forming in the chromosome and decatenates the two chromosomes that result from replication [30,61]. Even though these DNA gyrase and Topoisomerase IV are essential for cell survival, they have the potential to fragment the genome, and this is the characteristic that QNs use to destroy the bacterial cell [32].

FQNs exert their action by binding to one or both target enzymes and the DNA, stabilizing the cleavage complexes [31]. Most commonly, DNA gyrase represents the main target of QN in Gram-negative bacteria. In contrast, Topoisomerase IV represents the main target of QN in Gram-positive bacteria, with DNA gyrase being the secondary target in this case [67]. The exact binding of the (F)QNs to the target enzymes is partially elucidated. X-ray crystallography facilitated the discovery of the localization of the amino acids involved in the F(QN)–target interaction. These are located near the active-site tyrosine, involved in DNA breakage [31]. More detailed studies have been conducted to investigate the active site of the Topoisomerase IV–DNA cleavage complex for *Streptococcus pneumoniae* with new 7,8-bridged FQNs. The new 7,8-bridge compounds have proven antibacterial activity and offer an alternative to design new FQNs substituted on the C1, C7, and C8 positions to increase activity against resistant bacteria [68].

**Figure 3:**
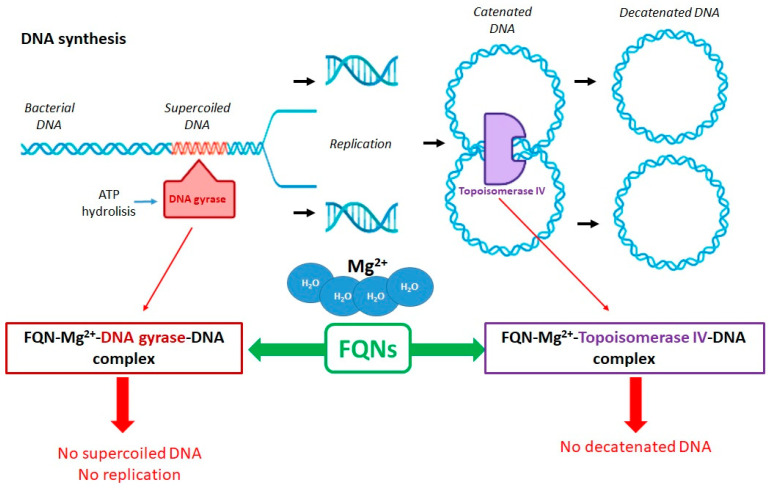
Role of DNA gyrase and Topoisomerase IV in the FQNs’ mechanism of action (adapted with permission from Ref. [25]).

The drug is intercalated between the DNA substrate and the enzyme. Interestingly, FQNs have a greater affinity for enzyme–DNA complexes than enzymes. The structural model of the drug–enzyme–DNA complexes has been discovered using X-ray crystallography. An essential part of connecting the FQN and the enzyme is the presence of a noncatalytic magnesium ion (Mg^2+^) coordinated with four water molecules and the C3/C4 FQNs’ carbonylic oxygens. Two water molecules coordinated with Mg^2+^ interact with the residues in the GyrA subunit of the enzyme. The magnesium ion is essential for forming a bridge between the enzyme and the drug. Interactions between the GyrB subunit and the C7 substituent of the FQN are also crucial for binding. Once the big complexes are formed (drug–Mg^2+^–enzyme–DNA), the enzymes become toxic to the cell, with the replication and transcription processes being blocked (Figure 3) [5,32,33].

However, the FQNs or the complexes they form do not kill the bacterial cell alone, especially since the genesis of complexes is reversible. There are a few critical factors that lead to either a slow death or to a rapid one.

- The slow death is caused by the unprocessed complexes that block replication and transcription;
- The immediate death occurs when the complexes are processed (by dissociation of the gyrase subunits or by removal of the gyrase from the DNA). In this case, the cell is killed due to the fragmentation of the chromosome, which results when the broken DNA is not repaired.

Additionally, more DNA breaks are caused by an accumulation of reactive oxygen species induced by damaged DNA and possibly by the cleavage complexes [5,32,33].

### 3.4. Indications, Spectrum of Activity, and Pharmacokinetics Data

Therapeutically relevant approved FQNs in the United States (US) and the European Union (EU) and their antimicrobial spectrum and indications [10,18,22,23,69,70,71,72,73,74,75,76,77,78,79,80,81,82,83,84] are presented in Table S1 (Supplementary Materials). The representatives from the first generations were used mainly in treating urinary tract infections caused by Gram-negative bacteria [9,26]. The second-generation representatives have expanded this utility spectrum to include the respiratory, urogenital and gastric tract, bone and joint infections, septicemia and surgical infections, some *Staphylococcus* spp., and venereal diseases [18,70,71]. Moreover, the representatives from the second generation have longer half-lives, less protein binding, and improved activity on Gram-negative bacteria [26]. Third-generation FQNs have markedly enhanced activity. Finally, the fourth generation is indicated in treating community-acquired pneumonia, skin and skin structure infections, bacterial conjunctivitis, and otitis externa [10,20,23,74,75,76,77,78,83,84]. The usual doses and indications of the most used antibacterial QNs are summarized in Table 2.

In addition to the FDA and EMA approvals, a few representatives are approved only in some states:

- Balofloxacin, third generation—approved in South Korea (2001) [104];
- Prulifloxacin, fourth generation—approved in Japan (2002) [105];
- Sitafloxacin, fourth generation—approved in Japan (2008) [106], Thailand (2012) [107];
- Nemonoxacin, fourth generation—approved in Taiwan (2014) [108];
- Zabofloxacin, fourth generation—approved in South Korea (2015) [109].

The pharmacokinetic properties of the several QNs are listed in Table 3. Some representatives have the potential to be incorporated into dual antibiotic hybrids.

### 3.5. Aspects to Be Considered Regarding the Inclusion of FQNs in Hybrid Compounds

When designing a hybrid, a few aspects must be balanced when choosing an FQN derivative as one of the components. One of the main disadvantages of this therapeutical class is the occurrence of side effects/adverse reactions [83,86,87,88,101,120,121,122,123,124,125,126,127]. Many representatives have been approved for human and veterinary use. Unfortunately, some have been withdrawn due to severe side effects [16,20,128]. The most common side effects of (F)QNs are related to the musculoskeletal and peripheral nervous system (e.g., tendinitis, tendon rupture, muscle weakness, muscle pain, joint pain, and joint swelling), the central nervous system (e.g., anxiety, depression, hallucinations, and confusion), and other body systems (e.g., worsening of myasthenia gravis, skin rash, sunburn, abnormal heart beat, and diarrhea) [129]. A more detailed approach to the main side effects of FQNs is presented in Table S2 (Supplementary Materials).

However, some advantages counterbalance the potential side-effects that might have otherwise driven scientists to search for other options in addition to FQNs. This class of antibacterial agents has advantages such as the mechanism of action that confers a bactericidal effect, their effectiveness and potency, and slower development of antimicrobial resistance, especially for the newer representatives, because of their dual activity against both target enzymes [14,27,130].

In addition to the advantages of antibacterial activity, FQNs also have an advantage from a chemical point of view. Their structures are relatively easy to synthesize, thus offering the possibility of developing numerous potential derivatives with various advantageous particularities [9,13,14,74]. Furthermore, FQNs have excellent complexing properties with metal ions due to their chemical structure and can form combinations with other active molecules [30,131]. The advantages mentioned above most likely counterbalance any drawbacks of the potential side effects. For this reason, FQNs have been the target of numerous attempts at hybridization and the development of new antibacterial agents [58].

## 4. Antimicrobial Resistance

The resistance of microorganisms has appeared since the first antimicrobial was used [132]. Antimicrobial resistance is the ability of microorganisms (such as bacteria, viruses, fungi, or parasites) to resist the action of an antimicrobial agent. Antimicrobial resistance may be due to intrinsic resistance (when microorganisms are naturally resistant to the action of certain antibiotics) or acquired (due to the adaptation of microorganisms through genetic modification) [133,134,135,136].

It is essential to find out which mechanism underpins the resistance to learn how to combat this threatening phenomenon. Additionally, knowledge of the mechanisms involved helps in the design of new molecules of antimicrobials to overcome resistance. The general mechanisms of antimicrobial resistance are genetic (transfer of genes), mutations, target-mediated mechanisms, inactivation or modification of antimicrobial molecules, reduced uptake of antimicrobials, active efflux, and biofilms [137,138,139,140]. Another important aspect is the prudent use of antimicrobials by avoiding their misuse or overuse [141,142]. Research shows that antibiotic resistance may also occur independently of antibiotic exposure [143,144].

### 4.1. Highlights of the Most Resistant Bacteria Worldwide

According to the World Health Organization (WHO), the most resistant bacteria currently existing are divided into three categories according to how urgent the need to discover new antibiotics is (Table S3, Supplementary Materials) [145]. Out of these pathogens, some are resistant to FQNs. The mechanisms by which bacteria develop resistance to FQNs are alterations in target enzymes, altered drug permeation (both in Gram-positive and Gram-negative bacteria), and plasmid acquisition [146]. Over time, resistance to FQNs developed alongside researchers’ efforts to improve the molecules of this class [25,147,148].

### 4.2. The Development of Antibacterial Resistance over Time

Since the introduction of the first antibiotic in therapy, there have been different levels of interest in the antibiotic resistance phenomenon. Podolsky (2018) described five eras of response to antibiotic resistance. Between 1945 and 1963, when antibiotic resistance appeared to be controlled by the pharmaceutical industry, little effort was undertaken to combat this threat, mainly on a local scale. During 1963–1981, a growing concern arose, fueled by the discovery of bacterial resistance spread across strains or species through what we now know as plasmids [149,150]. Then, from 1981–1992, this threat was beginning to be approached from a more global perspective, raising awareness of the misuse of antibiotics on multiple levels. From 1992–2013, concerns over antibiotic resistance increased; this is a shared global problem that requires interventions spread across various sectors. Finally, from 2013 to the present, the burden of antibiotic resistance is still viewed with great concern while emerging infections with resistant pathogens continue to spread globally [151].

**Figure 4:**
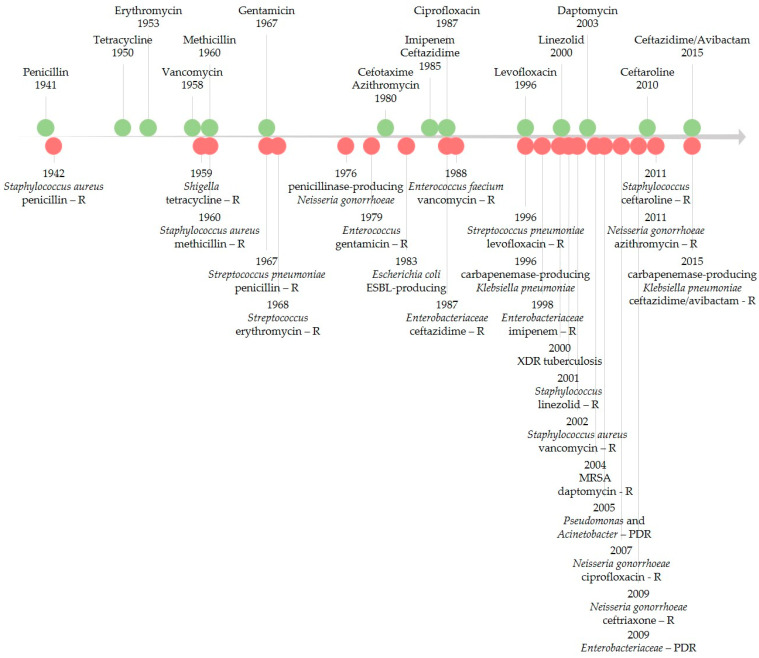
The evolution of identified resistance is in line with the introduction in the therapy of a few highlighted antibiotics [152,153].

Figure 4 illustrates the timeline of key points of antibiotic resistance occurrences based on early literature reports of resistance and reports of healthcare transmission or outbreaks [152,153,154]. FQNs were no exception for the development of antibacterial resistance [5,148,155]. Resistance to FQNs has arisen after widespread use in humans and animals [4,156]. Between 2001 and 2006, FQN-resistant *E. coli* isolates dramatically increased in the United Kingdom (from 6% to 20%). By 2010, it decreased to 17%, a phenomenon possibly linked to changes in prescribing [157]. For Enterobacteriaceae (e.g., *E. coli*), even higher QNs resistance rates were recorded worldwide. In 2015, in the US, reports showed the problematic fact that up to 30% of community-associated isolates were FQN non-susceptible [158]. As the figure highlights, antibiotic resistance is a never-ending phenomenon, unfortunately directly linked to the number of used antibiotics [159].

### 4.3. The Emergence of Resistance to Antibiotics Relatively Recently Introduced in Therapy

There have also been reports of resistance or possible mechanisms of resistance development to antibiotics relatively recently introduced in therapy (Table S4, Supplementary Materials) [160,161,162,163,164,165,166,167,168,169,170,171,172,173,174,175,176,177,178,179,180,181,182,183,184]. The leading causes of antibiotic resistance’s rapid emergence are overuse, inappropriate prescribing, and extensive agricultural use. Concerning the availability of new antibiotics, the economic and regulatory obstacles are mainly incriminated in hindering the development of these substances [160,185,186,187,188,189]. Improper or excessive use of antimicrobial agents accelerates the natural process of resistance [190]. Without effective antibiotics, the possibility of treating infectious diseases is endangered. Additionally, various medical procedures such as organ transplantation or major surgery could become even riskier. Antimicrobial resistance also impacts rising costs due to extended hospital stays and the need for longer-term intensive care [135].

### 4.4. New Mechanisms for Bacterial Resistance

Bacteria are constantly gaining resistance due to their genetic plasticity, suffering mutations frequently. They include new genes in their DNA relatively easily through transformation, transduction, and conjugation. These processes allow sharing of the resistance genes from a “gene carrier” bacteria to another. These mutations lead to multiple modifications in the cell and, in the end, to a form of resistance [133,191,192].

A good example is the resistance of *Bacteroides fragilis* to metronidazole. *Bacteroides fragilis* is an anaerobic colon resident, but it was found in many extraintestinal infections such as foot, brain, and abdominal infections. The resistance of *Bacteroides fragilis* is mainly correlated with nim genes in the chromosome or plasmid and multi-drug efflux pumps [193].

A complex mechanism of resistance is bacteria-forming biofilms. For example, *Pseudomonas aeruginosa* is a dangerous pathogen that manifests adaptive antibiotic resistance in addition to its existing resistance mechanisms such as efflux systems, antibiotic-inactivating enzymes, and decreased outer membrane permeability. Adaptive resistance is a response to environmental conditions, and it consists in forming a biofilm and existing in the form of persisting cells that tolerate the antibiotic. The biofilm is an aggregate of bacteria in a polymeric material. Living bacteria in the biofilm are more resistant to antibiotics due to the decreased permeability. In addition, the persisting cells in the biofilm are incapable of replicating in the presence of the antibiotic. Moreover, when the antibiotic is no longer present, they repopulate the biofilm and are responsible for the reactivation of chronic infections [194]. In this regard, some ciprofloxacin-nitroxide hybrids synthesized by Verderosa, A.D. et al. (2017) demonstrated the potential to overcome the resistance of biofilms to antimicrobials in two ways: stimulation of biofilm dispersal or direct cell killing [195].

On the other hand, the persistence of antibiotics is less understood nowadays. Eisenreich W. et al. (2022) addressed this phenomenon in a recently published review article. They proposed a new theory related to the persistence state of bacteria. So, in this state, bacteria become more susceptible to mutation-based antibiotic resistance [196].

### 4.5. Resistance to FQNs

There are a few reasons why bacterial resistance to FQNs develops. The dose and duration of administration of the drug are two essential factors. In addition, repetitive exposure and administration of low doses of FQN can enhance bacterial resistance, causing multiple mutations. Therefore, a critical aspect of avoiding bacterial resistance is maintaining a proper schedule of drug administration to ensure that the serum concentrations of FQN are higher than the minimum inhibitory concentration (MIC). Additionally, repeated use of the same agent should be avoided [197].

Resistance to FQNs occurs because bacteria use multiple mechanisms to adapt and survive when interacting with the drug [198,199]. One of the most used resistance mechanisms is the mutation of the genes that encode the type II Topoisomerases. This mechanism focuses on the alteration of the target site known as the quinolone resistance-determining region (QRDR) [130] and leads to a lower quinolone-binding affinity of the Topoisomerase enzymes [200]. Usually, concerning Gram-negative bacteria, FQNs affect the gyrase while in Gram-positive bacteria, FQNs target the Topoisomerase IV [197]. These mutations allow the bacteria to adapt after contact with the FQN [5]. So, as a result, it is considered that in Gram-negative bacteria, resistance occurs due to alterations in the DNA gyrase. In contrast, in Gram-positive bacteria, it is due to Topoisomerase IV mutations [201].

As DNA gyrase and Topoisomerase IV are cytoplasmic enzymes, achieving low cytoplasmic FQN concentrations is considered another bacteria solution that confers resistance [146]. Another mechanism includes mutations that reduce drug accumulation [197], such as downregulation of chromosome-encoded porins or increased drug elimination, by multi-drug efflux pumps [200]. The maintenance of low concentrations of FQN in the bacterial cells of Gram-positive bacteria results from the action of three efflux pumps, members of the major facilitator superfamily (MFS) of transporters [202]. One of them (NorA) is involved in the resistance development of hydrophilic FQN (e.g., norfloxacin). At the same time, the other two (NorB and NorC) are responsible for the resistance to hydrophilic and hydrophobic QNs (e.g., moxifloxacin, sparfloxacin). The efflux pumps are also present in Gram-negative bacteria, part of the transporters’ resistance nodulation-division (RND) superfamily [146].

However, unlike Gram-positive bacteria, where resistance results from active efflux transporters [33], Gram-negative bacteria have a structural advantage conferred by their double-membrane structure. The cell wall of Gram-negative bacteria acts as a barrier for hydrophilic molecules since the ability to infiltrate through the outer membrane is conditioned by the presence of porin proteins. Mutations that result in the downregulation of these proteins reduce cellular FQN accumulation as a consequence, especially that of the hydrophilic molecules [130,197,201].

Resistance mechanisms can also be encoded in mobile genes called plasmids [197], known as plasmid-mediated quinolone resistance (PMQR) genes [203]. Some of them encode transporters that can export drugs such as FQNs. Plasmids’ efflux pumps are essential in supporting the resistance to FQNs because they can remove the drug from the bacterial cell [130]. Additionally, to protect the bacterial cell from the FQN effect, they can encode topoisomerase-binding proteins or a modified enzyme that decreases FQN activity [200].

The activity of older FQNs has been studied to enhance the properties of new compounds regarding the installation of bacterial resistance. It was concluded that with newer FQNs, the bacterial resistance installs less rapidly because of their dual activity against DNA gyrase and Topoisomerase IV [14,130]. Furthermore, since both targets are equally affected, it would be less likely to elicit mutational resistance [197].

**Figure 5:**
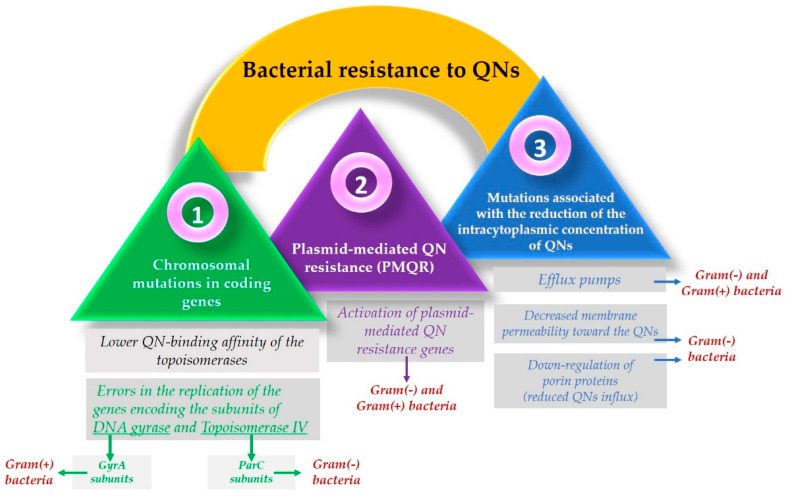
The main bacterial resistance mechanisms to QNs [60,199].

Specific structural changes to FQNs have been made to achieve this more complex targeting. This is the example of some fourth-generation representatives of FQNs; they are the result of improving the old FQN’s structure by adding a methoxy radical at the C8 position. This structural change can be found in moxifloxacin and gatifloxacin. In addition to this modification, gatifloxacin has a methyl group on the piperazinyl ring and moxifloxacin has a bicyclic ring in position C7. These structural changes were thought to be responsible for the mechanism of action targeting both DNA gyrase and Topoisomerase IV in Gram-positive bacteria. However, the exact reason these compounds act like this is still unclear. Initially, it was considered that their C8 methoxy group was the trigger for this action. Moreover, it was concluded that this type of targeting was not just the result of the methoxy group because delafloxacin, another FQN, does not possess this radical and is also responsible for the exact targeting [5,201]. Furthermore, delafloxacin is a more acidic FQN and is consequently more susceptible to deprotonation at a neutral pH. Therefore, as a consequence, delafloxacin shows an improved cellular uptake in acidic conditions [5]. The mechanisms involved in the development of bacterial resistance to QNs are illustrated in Figure 5.

## 5. Antibiotic Hybrids

### 5.1. Antibiotic Hybrids as Tools against Antimicrobial Resistance

Spizek and Havlicek (2015) summarized five strategies that could be used to fight the global phenomenon of antibiotic resistance. The first is the development of vaccines that target resistant bacterial strains; secondly, the discovery of new antibiotics (from conventional and less conventional sources); and thirdly, the discovery of new genes that specify the biosynthesis of antibiotics. The fourth strategy is the use and possible adaptation of natural compounds that have fallen out of interest in the present. The last proposed strategy is the discovery of new antibiotic targets [204]. The search for new compounds that possess either natural or synthetic antibiotic effects that are aimed at either traditional or more recent targets still receives interest from scientists [205,206]. Finally, an emerging strategy in the fight against antimicrobial resistance is the development of antibiotic hybrids. Some authors define antibiotic hybrids as “a synthetic construct of two or more pharmacophores belonging to an established agent known to elicit a desired antimicrobial effect” (Domalaon et al., 2018) [207].

The term “hybrid” suggests a two-component molecule with biological activity that retains the activity of the individual components after hybridization, acting synergistically. For example, hybrid drugs that incorporate two active compounds into a single molecule could be used to expand the biological activity and prevent the development of bacterial resistance [131]. Molecular hybridization combines the pharmacophore groups of different bioactive substances to produce a new hybrid molecule with complementary activities and/or multiple pharmacological targets and/or counterbalancing side effects compared to the original molecules. Over the last years, there have been numerous attempts at obtaining and testing these hybrids against various bacterial strains, with many proving successful [19,58,131,208,209,210,211,212,213,214,215,216,217,218,219,220,221].

QNs and FQNs are good candidates for hybridization due to their chemical structure, which facilitates linkage with many other active compounds [58]. In addition, other advantages that make FQNs promising for incorporation in antibacterial hybrids are the mechanism of action that confers a bactericidal effect, their effectiveness and potency, and the slower development of antimicrobial resistance [14,27,130].

#### Prodrug versus Hybrid Comparison

A prodrug is a pharmacologically inactive molecule converted in vivo into active forms by enzymatic or chemical reactions. By designing a prodrug, the pharmacokinetic properties of the active drug (such as bioavailability, absorption, and permeability) can be modified without affecting its pharmacological activity. Prodrugs can be classified into three categories: (1) carrier-linked prodrugs (an active drug linked to a pro-moiety), in which the active drug is released after an enzymatic or chemical reaction by which the moiety is removed; (2) bio-precursor prodrugs (the active drug is modified at the molecular level), where oxidation or reduction reactions modify the structure and release the active drug; and (3) double prodrugs (two biologically active drugs are linked in a single molecule), where the linkers between the two drugs can be cleaved by different mechanisms to release the component molecules [222].

Prodrug design has been used for (F)QNs to improve their physicochemical and pharmacokinetic properties (e.g., water solubility, lipophilicity, absorption, bioavailability) [41,42,223,224]. Some examples of the obtained FQNs’ prodrugs are alatrofloxacin (mesylate salt, a prodrug of trovafloxacin) [7,225,226], bisphosphonated fluoroquinolone esters [7,225,227], polyester prodrugs of norfloxacin [7,225,228], cellulose ether derivatives of ofloxacin [7,225,229], moxifloxacin conjugated with hydrophilic cellulose ethers [7,225,230], alalevonadifloxacin (*L*-alanine ester prodrug of levonadifloxacin), and N-Acylated ciprofloxacin derivatives [7,43,115,225].

Antibiotic hybrids represent two covalently linked pharmacophores with different mechanisms of action [231]. The design of hybrids (antibiotic–antibiotics or antibiotic–adjuvant) aims to surmount the resistance mechanisms for either or both drugs. The combination with an adjuvant helps by increasing the access to the target site or augmenting the primary antibiotics’ efficacy [7,225].

### 5.2. Structural Considerations regarding Antibiotic Hybrids

**Figure 6:**
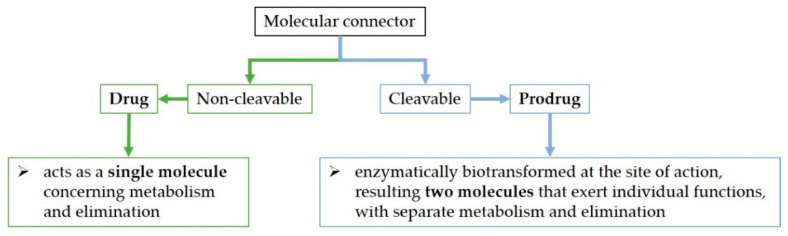
The drug versus prodrug approach.

Although molecules can be directly joined in hybrids, a molecular connector can bind the active molecules together through a covalent bond. The bond can be cleavable or non-cleavable. A hybrid with a cleavable connector would be enzymatically biotransformed when reaching the site of action (the hybrid prodrug approach—mutual prodrug) [222,232]) whilst the non-cleavable linker would remain intact for the duration of its time course in the body (the hybrid drug approach) (Figure 6) [7,207,225]. For example, the valine–citrulline linker is cleavable in the DSTA4637S hybrid [233]. On the other hand, the hybrid named cefiderocol contains a non-cleavable linker [207,234].

Regarding an antibiotic hybrid prodrug, the compound is cleaved into two molecules that exert individual functions, with separate metabolism and elimination. On the other hand, the antibiotic hybrid possessing the non-cleavable connector acts as a single molecule concerning metabolism and elimination [7].

**Figure 7:**
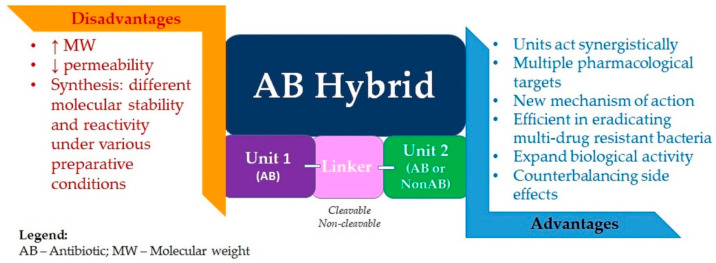
Advantages and disadvantages of antibiotic hybrids.

Compared to antibiotic combination therapy, antibiotic hybrids would suppress resistance with a single molecular agent, having a single pharmacokinetic profile, while also overcoming the possibility of noncomplementary pharmacodynamics. There is also the premise that hybrid drugs could affect the bacterial strains that are intermediately susceptible or resistant to one of the drug components. Moreover, although uncertain, there is the possibility of retaining antibacterial potency even against pathogens with resistance or intermediate susceptibility to both drug components. Supplementary physicochemical properties lacking in the original molecules could be imparted to the hybrid. It could translate into enhanced efficacy or even a new mechanism of antibacterial action for the obtained hybrid (Figure 7) [7,207].

The concept of an antibiotic hybrid is notably more widespread in the literature than the concept of a prodrug hybrid. An essential challenge in the hybrid prodrug approach is finding a linker specifically cleavable by bacterial enzymes and resistant to human metabolic enzymes. However, both methods require great efforts for synthesis due to the components’ different molecular stability and reactivity under various preparative conditions. Another challenge of designing hybrid drugs is imposed by the characteristic high molecular weight (>600 g/mol) of the resulting molecule; synthesizing agents able to penetrate the dual membrane of Gram-negative bacteria is reasonably difficult. However, several hybrid drugs, efficient in eradicating multi-drug-resistant Gram-negative bacteria and likely capable of delaying drug resistance onset, are currently in preclinical or clinical evaluation, thus bringing hope of a favorable prognosis for this strategy [207,235].

### 5.3. Obtained Hybrids with Antibiotics

Examples of antibiotic hybrids in various study phases are presented in Table 4. Many hybrids have been developed to fight Gram-negative bacterial infections [207]. A particular class combines antibiotics with siderophore-type molecules (e.g., cefiderocol) [234]. The siderophores act based on the “Trojan horse” strategy: bacterial iron uptake systems are used, and siderophores enter and destroy bacteria. More macrocycle–antibiotic hybrids are in various stages of development [233].

Additionally, hybrids that include FQNs are numerous and will be presented separately in the following section.

The design of new antibiotics must overcome the passage through the membranes of bacteria. Dual-acting antibiotic hybrids are promising agents to overcome drug resistance in multi-drug-resistant bacteria. However, the high molecular weight (over 600 g/mol) and pharmacokinetic differences of antibiotic hybrids are significant disadvantages for permeability and metabolism [207,259]. On the other hand, it seems that the molecular mass as a criterion for a drug-like compound (Ro5) needs to be updated. Many drugs or prodrugs violate one or even two Ro5 rules (e.g., cyclic peptide immunosuppressants, macrolide antibiotics, HIV protease inhibitors, tyrosine kinase inhibitors, antifungals, anti-cancers). Oral drugs “beyond the Ro5” (bRo5) seem to need a specific degree of flexibility to present aqueous solubility, transport through cell membranes, and target binding [260,261,262]. In 2020, 15 of the 26 drugs approved by the FDA (58%) violated one or more drug-likeness pharmacokinetic principles [263]. Therefore, we highlight that antibiotic hybrids cannot be discriminated against based on their high molecular weight without proper fundamental and clinical research. As an alternative, hybrids with antibiotic effects could also be used topically for treating various infections with multi-resistant pathogens. A good example is the hybrid TNP-2198 (Table 4).

Due to technological progress, computer-aided drug design (CADD) methods are beneficial for predicting new molecules with antibacterial activity and designing “hybrid” molecules. Examples of discovered compounds through CADD and bacteria on which they have potential action have been presented by Jukič and Bren (2022) in their review article [264].

### 5.4. Hybrids with FQNs

Hybridization of FQNs with other molecules (e.g., aminoglycosides, benzofuroxanes, oxazolidinones, etc.) produces candidates with synergistic antibacterial effect, activity on resistant bacteria, reduced toxicity, or other biological effects. To date, studies have been performed in which FQNs have been included in hybrids with various molecules, both other antibiotics or non-antibiotics (e.g., substances of the aminoglycoside class (ciprofloxacin-neomycin [216], moxifloxacin-tobramycin [211]), oxazolidinones (ciprofloxacin-linezolid [212]), or with benzofuroxan [217] and benzimidazole derivatives [219]) Table 5 comprises the antimicrobial activity of the hybrids presented in the following section.

#### 5.4.1. Antibiotic–Antibiotic Hybrids

**Figure 8:**
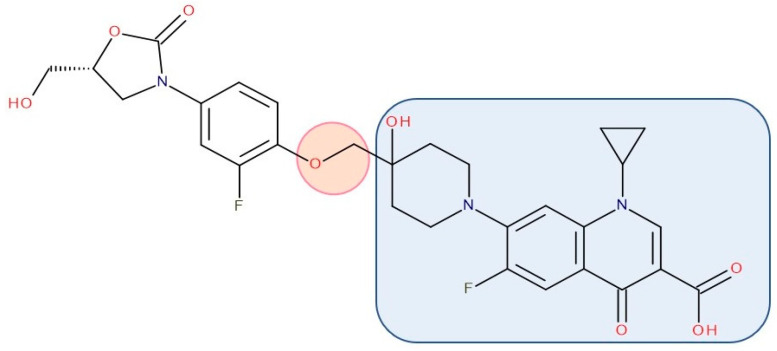
Antibiotic–antibiotic hybrid containing an FQN: tedizolid derivative–linker–ciprofloxacin derivative (Cadazolid); the linker is highlighted with the orange circle while the QN/FQN unit is highlighted by the blue rectangle [236].

**Figure 9:**
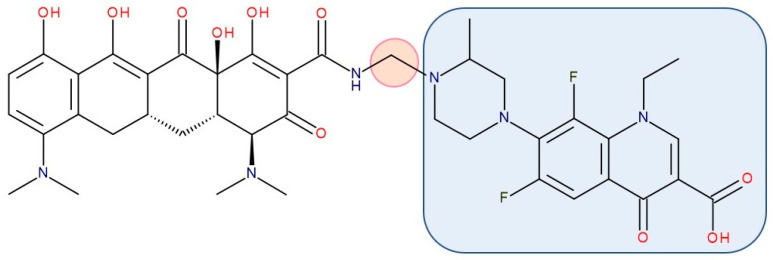
Antibiotic–antibiotic hybrid containing an FQN: minocycline–linker–lomefloxacin; the linker is highlighted with the orange circle while the QN/FQN unit is highlighted by the blue rectangle [267].

**Figure 10:**
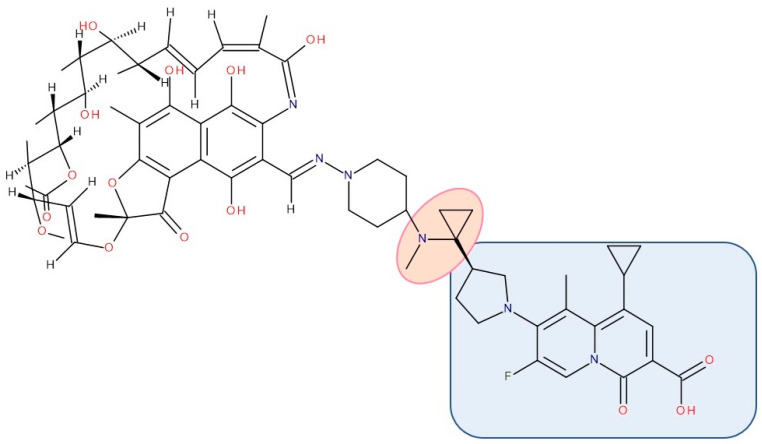
Antibiotic–antibiotic hybrid containing an FQN: rifampicin derivative–linker–ciprofloxacin derivative; the linker is highlighted with the orange circle while the QN/FQN unit is highlighted by the blue rectangle [268].

**Figure 11:**
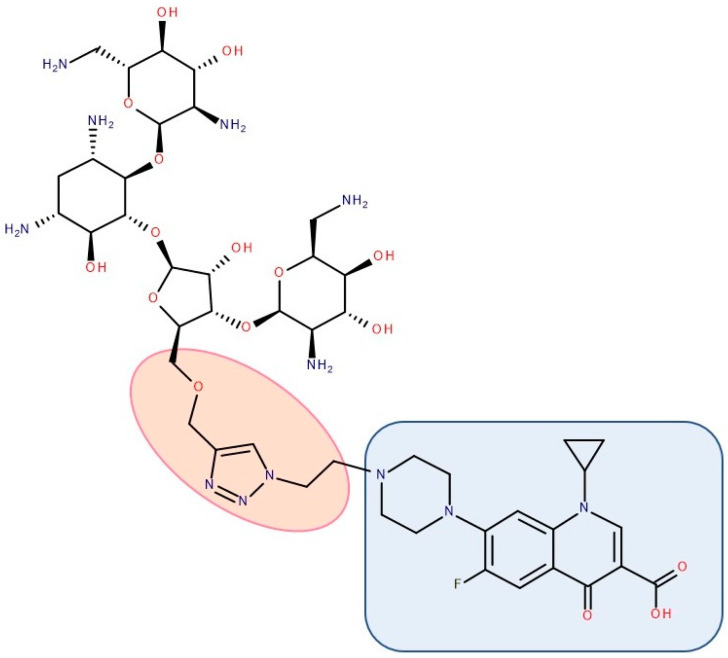
Antibiotic–antibiotic hybrid containing an FQN: neomycin B–linker–ciprofloxacin; the linker is highlighted with the orange circle while the QN/FQN unit is highlighted by the blue rectangle [216].

**Figure 12:**
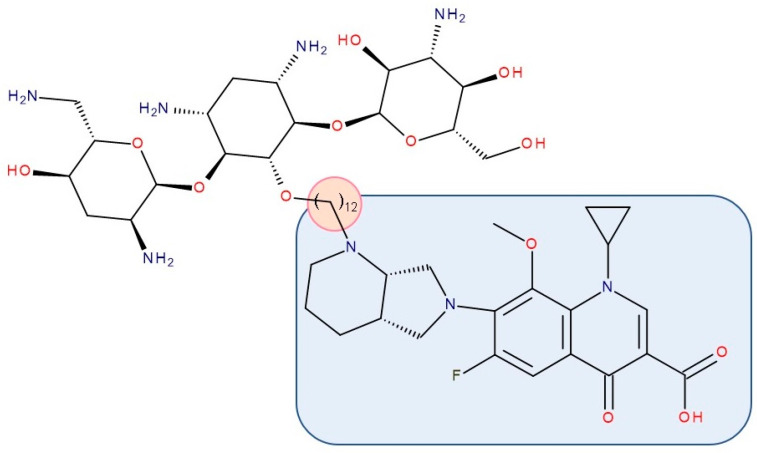
Antibiotic–antibiotic hybrid containing an FQN: tobramycin–linker–moxifloxacin; the linker is highlighted with the orange circle while the QN/FQN unit is highlighted by the blue rectangle [211].

**Figure 13:**
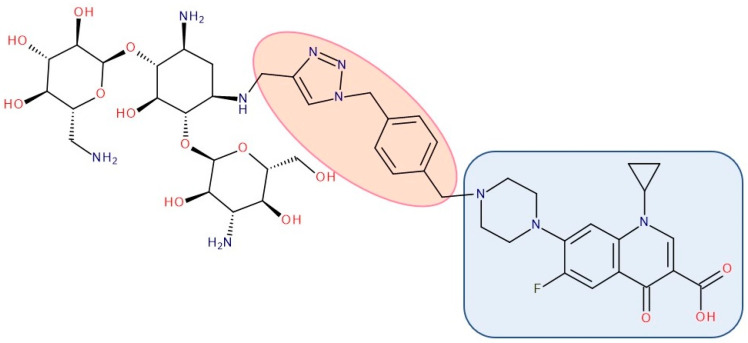
Antibiotic–antibiotic hybrid containing an FQN: kanamycin A–linker–ciprofloxacin; the linker is highlighted with the orange circle while the QN/FQN unit is highlighted by the blue rectangle [210].

Most published FQN hybrids present a linker between the two parent molecules. The two antibiotic molecules’ connectors differ from study to study (a carbon unit or more or diverse chemical elements). Figure 8, Figure 9, Figure 10, Figure 11, Figure 12 and Figure 13 illustrate such examples (the linker is highlighted with the orange circle while the blue rectangle highlights the FQN unit) [7,259]. Each research group probably selected the most successful linker and the simplest way to obtain an antibiotic hybrid. Apart from the “cleavable/non-cleavable” classification, the connectors have not yet been classified according to other criteria.

The most advantageous way of binding is to the radical in position 7 of the structure of FQNs, responsible for the antimicrobial potential and pharmacokinetic properties; thus, the groups responsible for binding to the bacterial target enzymes remain unaffected. Among the binding possibilities in the structure of hybrids is the formation of Mannich bases, between tetracyclines, formaldehyde, and the secondary amino group (piperazine) of FQNs [267].

A series of quinolone–fluoroquinolone hybrids were synthesized through benzotriazole chemistry. The C7 positions (the piperazine ring) of ciprofloxacin and norfloxacin and the amino acid linkers were targeted to obtain the final compounds. The obtained hybrids presented antibacterial properties that were comparable with the parent compounds [265].

Various researchers have synthesized ciprofloxacin derivatives using N-alkylations of the C-7 chain to increase the lipophilia and antibacterial potential [266,278]. The combination with **oxazolidinone** can be achieved by a bridge linking an FQN to the pharmacophore groups of the oxazolidinone derivative [212]. One of the promising hybrids that reached the phase 3 clinical stage is cadazolid. Cadazolid is a hybrid that contains structural elements of an oxazolidinone with an FQN moiety with significant activity against *Clostridium difficile* (Figure 8) [236,279,280].

Gordeev et al. (2003) synthesized several compounds that incorporated pharmacophore structures of FQNs and **oxazolidinones** and demonstrated superior potency to linezolid against Gram-positive and Gram-negative bacteria, even for linezolid- and ciprofloxacin-resistant strains of *Staphylococcus aureus* and *Enterococcus faecium*. The mechanism of action combined the inhibition of protein synthesis and DNA gyrase and Topoisomerase IV [212].

Representatives from the **tetracyclines** class (tetracycline, oxytetracycline, and minocycline) were combined with the secondary amino (piperazine) function of FQNs (norfloxacin, lomefloxacin, ciprofloxacin, and gatifloxacin) by Sriram et al. (2007). The results revealed anti-HIV and antitubercular activities, which were most significant for one of the compounds (minocycline-lomefloxacin derived—Figure 9), making it a promising candidate in treating patients with HIV-1 and co-infected with *Mycobacterium tuberculosis* [267].

CBR-2092 combines **rifampicin** and QNs in a hybrid antibiotic structure (Figure 10). Studies showed increased bactericidal activity against *Staphylococcus aureus* exhibited by CBR-2092, superior to that of rifampicin, moxifloxacin, or the combination of rifampicin and moxifloxacin. Furthermore, it is retained against strains that are intermediate or resistant to rifampicin or quinolone. Additionally, this hybrid prevented the development of resistance and was not a substrate for *Staphylococcus aureus* efflux pumps (NorA or MepA) [268]. Further studies showed that CBR-2092 exhibited a similar potency to rifampicin as an inhibitor of RNA polymerase, inhibited DNA gyrase and DNA Topoisomerase IV, and maintained activity against a variant commonly resistant to quinolone. Furthermore, CBR-2092 showed effects similar to rifampicin on RNA synthesis in strains susceptible to rifampicin and quinolone-like effects on DNA synthesis in strains resistant to rifampicin [281].

In a recent study, **kanglemycin A** (a rifampicin analogue) was linked to nine FQNs to obtain hybrids with superior antibacterial activity. Kanglemycin presents a dimethyl succinic acid moiety as an offering chemical group in synthesizing antibiotic hybrids. The activity of the synthesized hybrids linked to the acid group versus synthesized hybrids linked at the compound’s naphthoquinone ring system was compared. These have been proven to be determinants of the biological activity of the hybrids [282].

A series of hybrids with ciprofloxacin (FQNs) and **neomycin** (aminoglycoside) was synthesized by Pokrovskaya et al. (2009) (Figure 11). The antibacterial activity of most of the synthesized compounds was significantly higher than that of neomycin, in particular for Gram-negative bacteria and MRSA. Moreover, they overcame the most common types of aminoglycosides-associated resistance. When treated with the ciprofloxacin–neomycin hybrid, a significant delay in resistance formation against Gram-negative (*Escherichia coli*) and Gram-positive (*Bacillus subtilis*) bacteria was observed for the mixture of the two drugs or each drug separately. The hybrids’ mechanism of action could inhibit protein translation similar to or better than neomycin. Most importantly, they inhibited DNA gyrase and Topoisomerase IV up to 32-fold more than ciprofloxacin, proving a dual mechanism of action characteristic of hybrids [216].

**Azithromycin** and quinolone substructures were conjoined to preserve pharmacophores from both molecules; some obtained representatives showed an improved potency compared to azithromycin against Gram-positive and Gram-negative pathogens. Moreover, they maintained activity against macrolide-lincosamide-streptogramin-resistant strains of *Streptococcus pneumoniae* and *Streptococcus pyogenes*. Furthermore, they displayed increased potency over azithromycin and telithromycin against the Gram-negative *Haemophilus influenzae* [269].

Gorityala et al. (2016) used ciprofloxacin and moxifloxacin to synthesize conjugates with **tobramycin** (aminoglycoside) (Figure 12). Long carbon chains were used to link the compound molecules. The antibacterial properties were evaluated. In the synthesized series, among some hybrids that exhibited weak antibacterial effects, two of the hybrids showed good antibacterial effects against multi-drug-resistant strains of *Pseudomonas aeruginosa*. These conjugates destabilized the membrane and inhibited DNA gyrase A and Topoisomerase IV better than the original FQN and reduced efflux. The effect of the aminoglycoside (inhibition of protein translation) was reduced. However, it was observed that the development of bacterial resistance was delayed [211]. The ciprofloxacin–tobramycin hybrid was the first to be electrochemically characterized [283].

The emergence of resistance in *Escherichia coli* was evaluated for combining ciprofloxacin and **neomycin B** (aminoglycoside) compared to a hybrid drug obtained from the two antibiotics. The hybrids were synthesized, containing different linkers. For example, an aromatic triazole linker or hydroxyl group-containing aliphatic triazole linker united ciprofloxacin and neomycin B. The authors found that the bacterial populations grown in the presence of the hybrid developed less resistance than those produced in an equimolar mixture of the components. Furthermore, it was found that the ciprofloxacin part of the hybrid was responsible for the inhibition of bacterial growth while the neomycin B part limited resistance mediated by efflux [270]. A series of hybrids composed of ciprofloxacin (FQN) and **kanamycin A** (aminoglycoside) (Figure 13) were synthesized by Shavit et al. (2017) and showed superior activity against Gram-negative bacteria. These hybrids delayed the emergence of resistance for strains of *Escherichia coli* and *Bacillus subtilis* compared to the 1:1 mixture of the two antibiotics [210].

Most of the FQN antibiotic hybrids targeted in the manuscript are studied or are under study regarding their biological effects in vitro [211,212,216,265,266,267,268,269,270,270,278,281,282].

If the antibiotic hybrid contains a cleavable linker, there will be a high chance that the adverse reactions of the hybrid will be those of the FQN unit. However, in the case of a non-cleavable linker, it is possible to reduce the side effects of the FQN unit. Cadazolid is an FQN antibiotic hybrid in clinical phase 3 [236]. Seiler P. et al. (2019) reported that treatment with cadazolid did not lead to one potential side effect, namely the appearance of vancomycin-resistant enterococci, when treating *Clostridium difficile* infection. Therefore, cadazolid is a promising antibiotic alternative to vancomycin for treating *Clostridium difficile* infection [284,285]. Currently, few data are published concerning adverse reactions of hybrids with FQNs.

#### 5.4.2. Antibiotic–Non-Antibiotic Hybrids

**Figure 14:**
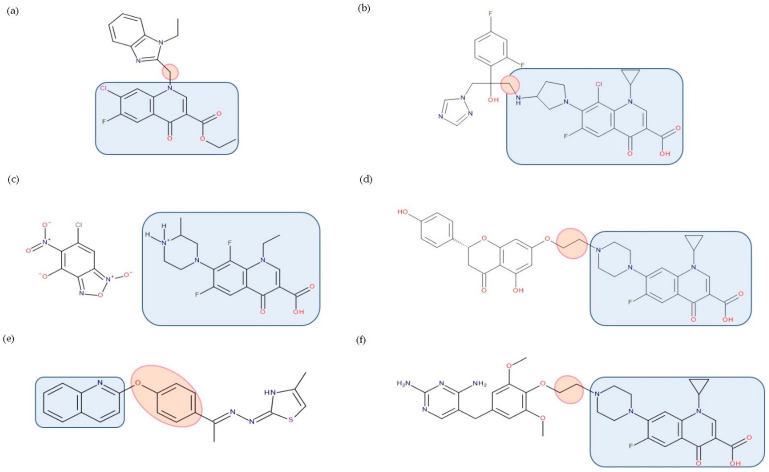
Examples of antibiotic–non-antibiotic hybrids containing an FQN; the linker is highlighted with the orange circle while the QN/FQN unit is highlighted by the blue rectangle: (**a**) benzimidazole derivative–linker–quinolone derivative (esther form) [219]; (**b**) triazole derivative–linker–clinafloxacin [286]; (**c**) benzofuroxan derivative–lomefloxacin [217]; (**d**) naringenin–linker–ciprofloxacin [207,273]; (**e**) thiazole derivative–linker–quinolone [287]; (**f**) trimethoprim–linker–ciprofloxacin [207,277].

Additionally, various hybrids of FQNs with different active substances were synthesized to broaden the antimicrobial spectrum, presented in detail below (Figure 14).

Durcik M. et al. (2021) designed and synthesized new hybrids of ciprofloxacin that can interact with the GyrA- and GyrB-binding sites of the target enzyme DNA gyrase. These new compounds demonstrate good activity against *Escherichia coli* and *Klebsiella pneumoniae*. In addition, without extensive efflux, some hybrids delayed or prevented the emergence of bacterial resistance [271].

**3-Arylfuran-2(5H)-one**

An array of covalently linked hybrids between FQNs and a tyrosyl-tRNA synthetase (TyrRS) inhibitor (3-arylfuran-2(5H)-one) was synthesized. Some hybrids displayed activity against both Gram-negative and Gram-positive resistant bacteria. A resulting hybrid of ciprofloxacin exhibited significantly greater potency against MDR *Escherichia coli* (MIC50—0.11 μg/mL) than the parent FQN (MIC50—5.65 μg/mL, for ciprofloxacin) [272]. This hybrid also displayed a dual mode of action (in vitro), having a more remarkable ability to inhibit DNA gyrase than ciprofloxacin and similar TyrRS inhibitory activity to that of the parent compound [207,272].

**Benzimidazole**

A series of hybrids between quinolone derivatives and benzimidazole was synthesized by Wang YN et al. (2018). One of the compounds showed remarkable activity against the resistant strains of *Pseudomonas aeruginosa* and *Candida tropicalis*. It also caused a decrease in the resistance of *Pseudomonas aeruginosa* compared to norfloxacin [219].

**Benzofuroxane**

Chugunova et al. (2016) synthesized a series of FQN hybrids with benzofuroxane derivatives; some hybrids showed superior antibacterial activity on *Bacillus cereus* 8035 strains compared to the free FQN [217].

**Chlorhexidine**

Kowalczuk D. et al. (2021) obtained ciprofloxacin–bismuth(III)–chlorhexidine, a new hybrid that contains the bismuth atom as a linker. This new hybrid (metal complex) has potential in the local treatment of wounds. So far, the published data have focused on the structural characteristics of the obtained hybrid using spectroscopic methods [288].

**Flavonoids (naringenin)**

Another collection of hybrids among FQNs and phenolic flavonoids was obtained. The most compelling representative was between ciprofloxacin and naringenin, with significant activity against methicillin-resistant *Staphylococcus aureus* (MIC50—0.29 μg/mL), *Escherichia coli* (MIC50—0.71 μg/mL), and amphotericin B-resistant *Candida albicans* (MIC50—0.14 μg/mL) [273]. This example also backs up the dual mode of action theory, displaying significant inhibition of both the DNA gyrase (specific to ciprofloxacin) and efflux pump (specific to naringenin) [273,289].

**1,3,4-Oxadiazole**

In a recent study, 1,3,4-oxadiazole derivatives were linked to the piperazine ring of ciprofloxacin or norfloxacin. Hybrids of the two FQNs with activity against Gram-positive bacteria were obtained. The molecular docking study revealed a high binding affinity for the hybrids 4 c for Topoisomerase IV with a minimum binding energy [274].

**Sulfonamides**

Nineteen novel ciprofloxacin-sulfonamide hybrid molecules showed significant antibacterial activity. In addition to biological activity, the side effects of hybrids were also tested. The following were used as linkers: azide, acetamide, propionamide, and isopropionamide. The most active hybrids presented lower CNS adverse reactions and GABA expression compared to those that used FQN [275].

**Thiazole**

The thiazole structural fragment is known for its numerous biological effects in medicinal chemistry research. A series of two-substituted quinolines, including a thiazole moiety separated by a hydrophobic linker, were synthesized and tested against Gram-positive and Gram-negative bacteria. The best structural element for antibacterial activity was the 2,3-dihydrothiazole fragment near the electron-donating group on the nitrogen atom of thiazole and the methyl at the carbon of azomethine. The authors of this study used the model of FQN hybrids with other antibiotics or sulfonamides, keeping the quinoline nucleus in the newly synthesized hybrids [287].

**Triazole**

An array of clinafloxacin triazole hybrids was synthesized, and their antimicrobial and antifungal activity were evaluated. Most compounds showed similar or better activity against the tested strains (Gram-positive bacteria—four strains, Gram-negative bacteria—four strains, and fungi—two strains) compared to chloramphenicol, clinafloxacin, and fluconazole. Moreover, clinafloxacin triazoles displayed improved efficacy on methicillin-resistant *Staphylococcus aureus* than clinafloxacin [286].

Another example of FQNs–triazole derivatives is provided by the study performed by Ezelarab et al. (2018). The antifungal activity of a ciprofloxacin–azole hybrid was evaluated, revealing promising results (MIC 10.23 µg/mL, comparable to itraconazole 11.22 µg/mL). Moreover, this obtained hybrid can reasonably bind to the active site of the target (lanosterol 14-α-demethylase CYP51) [276]. Other hybrids with FQNs were synthesized (1,2,3-triazole-substituted ciprofloxacin and norfloxacin derivatives); antibacterial and antifungal activities were investigated in silico and in vitro [290].

The use of the quinolone nucleus in hybrid compounds to obtain antimicrobial activity is supported by a recent study in which hybrids with quinolone derivatives and triazole were obtained. Triazole-linked quinoline derivatives from 8-aminoquinoline presented promising activities against Gram-positive and Gram-negative bacteria and some fungi strains [291]. Other 1H-1,2,3-triazole-linked quinoline–isatin hybrids were recently synthesized by Awolade P. et al. (2021); these new hybrids are promising anti-breast cancer and anti-MRSA agents [292].

**N-substituted trifluoroacetimidoyl chlorides**

Darehkordi et al. (2011) used N-substituted trifluoroacetimidoyl chlorides to synthesize piperazinyl-quinolone derivatives. Out of the obtained compounds, two exhibited superior antibacterial activity against strains of *Escherichia coli*, *Klebsiella pneumoniae* (compared to ciprofloxacin), and *Staphylococcus aureus* (compared to vancomycin) [209].

**Trimethoprim**

Although trimethoprim is not used in single therapy as an antibiotic, it was targeted by the hybridization strategy with an FQN. Trimethoprim linked to ciprofloxacin (through the piperazine ring) yielded a hybrid (BP-4Q-002) with good activity against *Staphylococcus aureus* (MIC 0.5 μg/mL) and *Escherichia coli* (MIC 1 μg/mL). Against the *Staphylococcus aureus* strain NRS19 (resistant to ciprofloxacin (MIC for ciprofloxacin—32 μg/mL, MIC for trimethoprim—4 μg/mL, and MIC for the equimolar mixture—8 μg/mL)), this hybrid exhibited an MIC value of 1 μg/mL) [277]. The activity of BP-4Q-002 against the drug-resistant *Staphylococcus aureus* strain endorses the concept that hybrid drugs may be able to eradicate strains resistant or intermediately susceptible to one of the parent compounds. Another contribution to a fundamental hypothesis of hybrid drugs (claiming that further qualities are imparted to the hybrid, which is missing in the parent components or the equimolar mixture) could be highlighted by the reduction in the MIC in the case of BP-4Q-002 against the *Staphylococcus aureus* strain NRS19, compared to the MIC of just ciprofloxacin, trimethoprim, or an equimolar mixture of the two [207].

### 5.5. FQN Hybrids with Other Biological Effects

FQNs are being studied for numerous other biological effects [3].

Thus, in addition to the hybrids’ antibacterial properties, as highlighted above for the antifungal effect [219,273,276], quinolone hybrid compounds also showed anti-HIV [267], antifungal [276,286,290], antiplasmodic/antimalarial [293,294], and antitumor [295] potential.

A considerable number of quinolone-based derivatives were synthesized for their antiplasmodial activity to be evaluated. Some displayed promising antiplasmodial (in vitro) activity against chloroquine-sensitive, chloroquine-resistant, and multi-drug-resistant strains of *Plasmodium falciparum*. At the same time, some showed significant antiplasmodial (in vitro) and antimalarial (in vivo) activity [293,294].

N-4-piperazinyl–ciprofloxacin chalcone hybrids were synthesized, and their activity against various cancer cell lines and topoisomerase inhibitory activity were evaluated. The obtained hybrids exhibited significant inhibitory activity on Topoisomerase I and II while a few of the compounds displayed broad antitumor activity [295].

Additionally, nitric oxide (NO) photo-donor of ciprofloxacin and norfloxacin hybrids were synthesized by Fallica, A.N. et al. (2021) to study the potential anticancer effect. This study showed that some hybrids have intense antiproliferative activity on breast cancer cell lines (aggressive, refractory, and multi-drug-resistant cancer type) [296].

## 6. Future Research Direction of FQN Hybrids

The importance of FQNs in human health is well established through their utility in treating many infections. Nowadays, it is crucial to reduce the antimicrobial resistance to FQNs. The strategy to design hybrids of FQNs with other antibiotics or active molecules is a reliable alternative in the fight against this worldwide menace. Numerous hybrids of FQNs previously presented are in various stages of research targeting the antibacterial effect and other biological effects [3].

The continuous discoveries about the structure–activity relationship in the FQNs class and advances in computer-aided drug design (CADD) methods will contribute to the coming generations of antibiotic hybrids [264,297]. New derivatives of FQNs are being discovered and studied. For example, using CADD and SAR, studies found trovafloxacin derivatives with lower binding to plasma proteins [298]. CADD methods could predict new molecules with antibacterial activity and design “hybrid” molecules [264]. Predictive models in virtual screening for new antibacterial agents are now possible due to machine learning techniques. In addition, machine learning techniques could be helpful in the prediction of antibacterial resistance and its mechanisms [297].

## 7. Conclusions

Hybridization of FQNs with other antibiotics or active compounds has significant potential for antibacterial effects, especially for action on multi-drug-resistant bacterial strains. Hence, these complex molecules could expand the antibacterial activity and delay the onset of resistance by overcoming pathways in which the bacterium decreases its susceptibility to antibiotics, such as increasing membrane permeability and reducing the efflux from the bacterial cell. Antibiotic–antibiotic hybrids have essential advantages over combined antibiotic therapy. A key element of these hybrids is the linker, which can be cleavable or non-cleavable. Various hybrids with other active substances were also synthesized to increase antibacterial activity or identify new biological effects. Many of the FQN synthesized hybrids are in multiple stages of research, some of which are in advanced clinical trials. This strategy of obtaining new antibacterial agents brings added value to the range of active molecules with potential in the fight against the installation of bacterial resistance, a continuous global challenge. However, nowadays, the design of FQN hybrids represents an insufficiently exploited niche in the battle against bacterial resistance.

## Figures and Tables

**Table 1:** Essential structure–activity relationship aspects in the antibacterial QNs class.

Position on the Chemical Structure	Requirements and Possible Implications	References
2	Optimal is a hydrogen moiety; larger moieties may hinder the C3 and C4 positions.	[9]
3	A carboxyl group is required (essential for interacting with the DNA bases and DNA gyrase).	[5,9,31,32,33]
4	Oxo-(keto) moiety is required; essential for interacting with the DNA bases and DNA gyrase.	
6	Small moiety is required (optimal—fluorine); fluorine increases the potency by between 5- and 100-fold compared to any other potential halogen moiety.	[9]
1	It is involved in the pharmacokinetic properties and overall potency. A cyclopropyl moiety confers activity against Gram-negative bacteria. A 2,4-difluorophenyl substituent determines less potency but heightens activity against anaerobes (e.g., temafloxacin; it was withdrawn shortly after approval due to severe adverse reactions).	[9,34,35]
5	Specific radicals substituted at this position (-NH_2_, -CH_3_) may increase activity against Gram-positive bacteria.	[9,34]
7	It is involved in pharmacokinetic properties and the spectrum of activity. A five- or six-membered nitrogen heterocycle at this position improves the activity and pharmacokinetic profile. For example, amino pyrrolidine or an alkyl moiety determines enhanced activity against Gram-positive bacteria. On the other hand, piperazine determines better activity against Gram-negative bacteria.	[9,34]
8	It is involved in the pharmacokinetic properties and activity against anaerobic bacteria.	[9]

**Table 2:** Doses and therapeutic indications (US Food and Drug Administration (FDA) and European Medicines Agency (EMA) approved).

Compounds (Generation)	Usual Doses	Indications and Administration	References
Nalidixic acid (1st)	4 g daily (every 6 h); 7 to 14 days in acute infections, reducing after that to half this dose in chronic infections.	Uncomplicated urinary tract infections; Oral administration.	[18,20,70,79]
Norfloxacin (2nd)	400 mg twice a day (every 12 h); 3–7–21–28 days depending on the severity and nature of the infection.	Uncomplicated and complicated urinary tract infections; Acute or chronic prostatitis; Uncomplicated gonorrhea; Oral administration.	[18,21,70,85,86]
Ciprofloxacin (2nd)	250–500 mg (every 12 h); 7 to 14 days or more, depending on the severity and nature of the infection.	Uncomplicated and complicated urinary tract infections, pyelonephritis, sexually transmitted diseases, prostatitis, skin and tissue infections; Oral (as the hydrochloride or base) and parenteral administration (lactate), eye drops, eye ointment, or ear drops (as the hydrochloride).	[18,70,87]
Ofloxacin (2nd)	200–400 mg twice a day (every 12 h); 3 days to 6 weeks, depending on the severity and nature of the infection.	Similar to ciprofloxacin. In addition, *Chlamydia* or *Chlamydophila* infections include nongonococcal urethritis and mycobacterial infections (leprosy and tuberculosis); Oral (as a base) and parenteral administration (as a hydrochloride salt).	[18,70]
Pefloxacin (2nd)	400 mg twice daily (every 12 h); similar to norfloxacin.	Uncomplicated gonococcal urethritis in males, Gram-negative bacterial infections in the gastrointestinal system and the genitourinary tract; Oral and parenteral administration (as a mesylate salt).	[18,21,85,88]
Nadifloxacin (*topical use*) (2nd)	Twice a day as cream or ointment (1%).	Acne vulgaris and other skin infections; Topical use.	[45,70,71,89]
Levofloxacin (3rd)	250–500 mg (once or twice daily); 7 to 14 days, depending on the severity and nature of the infection.	Acute and chronic bronchitis, exacerbated forms, acquired pneumonia (nosocomial), and other susceptible infections, including tuberculosis; Oral and parenteral administration (as a hemihydrate); Ophthalmic use (0.5% ophthalmic solution).	[18,70,74,90,91]
Gatifloxacin (*ophthalmic use*) (3rd)	Day 1:1 drop every 2 h in the affected eye(s) while awake, up to 8 times Day 2 to 7:1 drop twice to 4 times daily in the affected eye(s) while awake.	Bacterial conjunctivitis, ophthalmic use (0.3% or 0.5% ophthalmic solution).	[92,93,94]
Moxifloxacin (4th)	Oral: 400 mg once a day; 5–10 days depending on the severity and nature of the infection; Ophthalmic administration: one drop in the affected eye 3 times daily for 7 days.	Sexually transmitted diseases, prostatitis, skin and tissue infections, acute and chronic bronchitis, exacerbated forms, acquired pneumonia (nosocomial), intra-abdominal infections, gynecological infections, bacterial conjunctivitis; Oral, parenteral, and ophthalmic administration (0.5%) as a hydrochloride salt.	[75,77,95,96,97]
Delafloxacin (4th)	Intravenous: 300 mg over 60 min, every 12 h; Oral: 450 mg every 12 h; 5 to 14 days.	Bacterial skin and skin structure infections; Oral and intravenous administration.	[23,98,99]
Besifloxacin (*topical, ophthalmic use*) (4th)	Ophthalmic administration: 1 drop in the affected eye 3 times daily, 4 to 12 h apart for 7 days.	Bacterial conjunctivitis; Ophthalmic suspension (0.6%).	[22,100,101]
Finafloxacin (*topical, ophthalmic use*) (4th)	Optic administration: 4 drops in the affected ear(s) twice daily for 7 days.	Acute otitis externa; Optic suspension (0.3%).	[83,102,103]

**Table 3:** Pharmacokinetic data of some representative QNs.

FQNs	Single Dose p.o. ^1^ (g)	Plasmatic Concentration (μg/mL)	Half-Life (Hours)	Binding to Plasma Proteins (%)	Elimination Route	References
Avarofloxacin	0.25	2	14	65	renal	[110]
Ciprofloxacin	0.2	0.8	4–6	20–50	renal, hepatic, feces	[13,18,111,112]
Delafloxacin	0.45	5.80–7.17	4.2–14.9	84	renal	[98,99,113]
Enoxacin *	0.20	1.0	5	40–60	renal, hepatic	[13,18,25,112]
Fleroxacin *	0.4	5.0	10–12	23	renal, hepatic	[25,114]
Gatifloxacin *	0.20	2.0	7.8	20	renal	[13,25,75,112]
Gemifloxacin *	0.32	1.6	6.9	60–70	renal and others	[13,25,75]
Grepafloxacin *	0.40	0.93	12	50	hepatic, renal	[13,25]
Lomefloxacin *	0.2	0.7	3–4	10	renal	[18,25,112]
Levofloxacin	0.50	6.2–8.7	6–7	24–40	renal	[13,18,111]
(Ala)Levonadifloxacin	1	16.5	4.5	85	-	[115]
Moxifloxacin	0.40	4.5	12	30–50	hepatic, renal	[13,75,112]
Nalidixic acid	1.00	20–40	6–7	93–97	renal	[13,18,112]
Nemonoxacin	0.5	7.02	15	16	renal	[116]
Norfloxacin	0.40	1.5–2	4–8	15	renal, hepatic, feces	[18,85,112]
Ofloxacin	0.20	1.5	4.5–9	32–40	renal	[13,18,112]
Pefloxacin	0.40	3.9–5.8	8–13	20–30	hepatic, renal, feces	[117]
Sparfloxacin *	0.40	1.1–1.3	20	40–50	renal, hepatic	[13,18,25,111,112]
Temafloxacin *	0.60	2.43	8	25	hepatic, renal	[13,25,118]
Trovafloxacin *	0.10	1.0	9.1	76–85	hepatic	[13,25,112]
Zabofloxacin	0.4	2.0	8.24–8.32	NA ^2^	NA ^2^	[109,113,116,119]

^1^ p.o.—oral administration; ^2^ NA—not available; * Withdrawn.

**Table 4:** Examples of antibiotic hybrids in various stages of development (AB—antibiotic, LK—linker, NAB—non-antibiotic, C—cleavable, NC—non-cleavable, UTI—urinary tract infection).

Type	Hybrid (Commercial Name)	Unit 1 (Class)	Linker	Unit 2 (Class)	Possible Indications and Dosage	References
**AB-LK-AB**	Cadazolid	Tedizolid (oxazolidinones)	NC	Ciprofloxacin (FQNs)	*Clostridium difficile*-associated diarrhea—Phase 1 clinical trial—single oral dose of 3000 mg	[207,236,237]
	TNP-2092 (CBR-2092)	Rifamycin (ansamycins)	NC	Ciprofloxacin derivative (FQNs)	Gastrointestinal and liver disorders—*Clostridium difficile* infection model—6.67 mg/kg, orally, 7 days, Acute bacterial skin and skin structure infection—Phase 2 clinical trial—300 mg intravenously, every 12 h	[238,239,240,241]
	Cefilavancin (TD-1792)	Vancomycin (glycopeptide antibiotics)	NC	THRX-169797 (cephalosporins)	Gram-positive complicated skin and skin structure infections—Phase 2 clinical trial—2 mg/kg/day, intravenously	[233,242,243,244,245]
	TD-1607	Vancomycin (glycopeptide antibiotics)	C	THRX-169797 (cephalosporins)	Infections with Gram-positive bacteria—Phase 1 clinical trials to evaluate the tolerability, safety, and pharmacokinetics—single escalating doses, intravenously	[233,246]
	TNP-2198	Rifamycin (ansamycins)	NC	Metronidazole	*Helicobacter pylori* infection (mouse model), *Clostridium difficile* infection (hamster model)—5, 15, and 45 mg/kg/day, orally, 5 days;bacterial vaginosis	[233,247]
	MCB-3681	Linezolid (oxazolidinones)	NC	Ciprofloxacin derivative (FQNs)	Infections with Gram-positive bacteria—multiple-dose phase 1 study—6 mg/kg body weight over 12 h for 5 days, intravenously	[248]
**AB-LK-NAB**	Cefiderocol (Fetroja)	Ceftazidime (cephalosporins)	NC	2-chloro-3,4-dihydroxybenzoic acid (catechol derivative; siderophore)	Complicated UTI and severe carbapenem-resistant Gram-negative bacterial infection—Phase 3 clinical trial—2 g intravenously over 3 h every 8 h for a period of 7 to 14 days, or 2 g every 6 h for participants with creatinine clearance >120 mL/min	[207,249,250,251,252]
	-	Ampicillin/ Amoxycillin	NC	Enterobactin (catecholate siderophore)	*Escherichia coli* Infections—microbiological assay	[253]
	-	Ampicillin	NC	Tetramic acid(s)	Gram-negative bacterial infections—microbiological assay	[254]
	DSTA4637S	4-Dimethylaminopiperidino-hydroxybenzoxazino rifamycin (ansamycins)	C	Thiomab human immunoglobulin G1 (IgG1) monoclonal antibody	*Staphylococcus aureus* infections—Phase 1 clinical trials—low-, intermediate-, and high-dose intravenous infusion	[233,255,256,257,258]

**Table 5:** The antimicrobial activity of QN/FQN hybrids (represented through MIC).

Type of Hybrid	Compound Code	Microorganism	MIC	Reference
QN-FQN	**10f**	*Staphylococcus aureus*	3.3 μM	[265]
	**10b**	*Streptococcus pyogenes*	7.8 μM	
	**11a**	*Salmonella typhi*	7.6 μM	
	**11b**		7.4 μM	
N-alkylations of the C-7 chain of QN	**7l**	*Mycobacterium tuberculosis* H37Rv and multi-drug-resistant *Mycobacterium tuberculosis*	0.09 μM	[266]
Oxazolidinone-FQN	**2, 5 and 6**	*Staphylococcus aureus* *Enterococcus faecium*	≤1 μg/mL	[212]
Tetracycline-FQN	**10**	*Mycobacterium tuberculosis*	0.2 μg/mL	[267]
Rifamycin-QN	**CBR-2092**	300 clinical isolates of staphylococci and streptococci	0.008–0.5 μg/mL	[268]
Aminoglycoside-FQN	**1i**	*Escherichia coli* (R477-100, ATCC 25922, AG100B, AG100A)	0.75–3 μg/mL	[216]
	**1q**		0.38–12 μg/mL	
Azithromycin-QN	**7f**	*Streptococcus pyogenes*	0.5 μg/mL	[269]
	**8f**		1 μg/mL	
	**7f**	*Haemophilus influenzae* B 0529	0.5 μg/mL	
	**8f**		0.5 μg/mL	
Aminoglycoside-FQN	**1**	*Staphylococcus aureus* and methicillin-resistant *Staphylococcus aureus*	1 μg/mL	[211]
		three *Pseudomonas aeruginosa* strains (including two gentamicin-resistant *Pseudomonas aeruginosa* strains)	4−8 μg/mL	
Aminoglycoside-FQN	**1m**	*Escherichia coli*	6.2 ± 0.7 μM (day 1) 30.3 ± 3.4 μM (day 17)	[270]
Aminoglycoside-FQN	**1b**	*Escherichia coli* (R477-100, 25922, AG100B, AG100A)	0.37–12 μg/mL	[210]
		*Bacillus subtilis*	1.5 μg/mL	
ATP-competitive inhibitors (for DNA Gyrase A and B)-FQN	**3a**	*Klebsiella pneumoniae*	0.5 µg/mL	[271]
		*Enterobacter cloacae*	4 µg/mL	
		*Escherichia coli*	2 µg/mL	
3-arylfuran-2(5H)-one-FQN	**11**	Multiple drug-resistant *Escherichia coli*	0.11 μg/mL	[272]
Benzimidazole-QN	**5b**	*Pseudomonas aeruginosa*	1 μg/mL	[219]
		*Staphylococcus aureus* and methicillin-resistant *Staphylococcus aureus*	8 μg/mL	
		*Klebsiella pneumoniae*	16 μg/mL	
Benzofuroxane-FQN	**4d**	*Bacillus cereus 8035*	0.97 μg/mL	[217]
Flavonoids (naringenin)-FQN	**7**	*Escherichia coli*	0.71 μg/mL	[273]
		*Bacillus subtilis*	0.062 μg/mL	
		*Staphylococcus aureus*	0.29 μg/mL	
		*Candida albicans*	0.14 μg/mL	
1,3,4-Oxadiazole-FQN	**4 b–d**	*Staphylococcus aureus*	≤0.125 μg/mL	[274]
Sulfonamide-FQN	**3a**	*Staphylococcus aureus*	0.324 μM	[275]
		*Escherichia coli* ATCC8739	0.025 μM	
	**3b**	*Staphylococcus aureus*	0.422 μM	
		*Escherichia coli* ATCC8739	0.013 μM	
Triazole-FQN	**11**	*Candida albicans*	10.23 µg/mL	[276]
Trimethoprim-FQN	**BP-4Q-002**	*Staphylococcus aureus*	0.5 μg/mL	[277]
		*Escherichia coli*	1 μg/mL	
		*Staphylococcus aureus* NRS19 (resistant to ciprofloxacin)	1 μg/mL	

## Supplementary Materials

The following are available online at https://www.mdpi.com/article/10.3390/pharmaceutics14081749/s1, Table S1: Antimicrobial spectrum and indications for the antibacterial (F)QNs for human use; the generation is mentioned in parentheses (Ref. = references). Table S2: Side effects/adverse reactions of the antibacterial (F)QNs. Table S3: Pathogens classified by WHO according to the urgency of antibiotic discovery need. Table S4: The emergence of resistance to newer antibiotics (Ref. = references).
