# Supplementary material for: Fluoroquinolones Hybrid Molecules as Promising Antibacterial Agents in the Fight against Antibacterial Resistance
Source: Pharmaceutics. 2022 Aug 22;14(8):1749. doi: 10.3390/pharmaceutics14081749 (PMC9414178; doi:10.3390/pharmaceutics14081749)
Supplement: Supplementary file 1 [file pharmaceutics-14-01749-s001.zip › pharmaceutics-1862090-supplementary.pdf]

pharmaceutics

*Review—Supplementary Materials*

# Fluoroquinolones Hybrid Molecules as Promising Antibacterial Agents in the Fight against Antibacterial Resistance

**Ioana-Andreea Lungu, Octavia-Laura Moldovan, Victoria Biriș and Aura Rusu**

**Table S1.** Antimicrobial spectrum and indications for the antibacterial (F)QNs for human use; the generation is mentioned in parentheses (Ref. = references).

| Antibacterial (F)QNs | Antimicrobial spectrum, indications | Ref. |
|---|---|---|
| Nalidixic acid (1st) | Urinary tract infections caused by Gram-negative bacteria (*Escherichia coli*, *Klebsiella*, *Enterobacter*, *Citrobacter*, and *Proteus* spp., *Shigella*, *Salmonella*, and *Providencia*).<br>Resistant pathogens: Strains of *Pseudomonas aeruginosa*, *Neisseria gonorrhoeae*, *Haemophilus influenzae*, Gram-positive cocci and anaerobes. | [1] |
| Pefloxacin (2nd) | Gram-negative organisms and staphylococci; respiratory tract, urogenital tract, bone and joint infections, septicemia and surgical infections. | [2] |
| Norfloxacin (2nd) | Gram-negative and Gram-positive aerobic bacteria.<br>Urinary tract infections caused by *Escherichia coli*, *Klebsiella pneumoniae*, *Enterobacter cloacae*, *Proteus mirabilis*, indole-positive *Proteus* spp., including *Proteus vulgaris*, *Providencia rettgeri*, *Morganella morganii*, *Pseudomonas aeruginosa*, *Staphylococcus aureus*, and *Staphylococcus epidermidis*, and group-D streptococci.<br>Generally not effective against anaerobic bacteria.<br>The fluorine atom facilitates the activity against Gram-positive organisms, while the piperazine moiety improves antipseudomonal activity. | [1] |
| Ciprofloxacin (2nd) | Bacterial gastroenteritis caused by Gram-negative bacilli (enteropathogenic *Escherichia coli*, *Salmonella* spp. (including *Salmonella typhi*), *Shigella* spp., *Vibrio* spp., and *Aeromonas hydrophilia*); respiratory tract infections (especially bronchitis and pneumonia caused by Gram-negative bacteria); infections of the skin, soft tissues, bones, and joints; uncomplicated and complicated urinary tract infections caused by Gram-negative bacteria; chronic infections characterised by renal tissue involvement; venereal diseases; postexposure treatment of inhalational anthrax. | [1] |
| Ofloxacin (2nd) | Similar to ciprofloxacin. | [1] |
| Nadifloxacin (2nd) | Gram-positive (e.g., *Staphylococcus aureus*, methicillin-resistant *Staphylococcus aureus* (MRSA), and *Staphylococcus epidermidis*) and Gram-negative (e.g., *Pseudomonas aeruginosa*) bacteria, including anaerobes (*Propionibacterium acnes*); topical use. | [3,4] |

| Antibacterial (F)QNs | Antimicrobial spectrum, indications | Ref. |
|---|---|---|
| Levofloxacin (3rd) | Antibacterial spectrum and indications similar to the racemate (ofloxacin). | [1,5–7] |
| Moxifloxacin (4th) | Community-acquired respiratory tract infections caused by typical and atypical respiratory pathogens, intracellular respiratory pathogens; skin and skin structure infections, intra-abdominal infections;<br>Gram-negative pathogens and anaerobes; *Mycobacteria* spp., *Legionella.* | [7–11] |
| Gemifloxacin (4th) | Acute exacerbations of chronic bronchitis, community-acquired pneumonia; *Streptococcus pneumoniae, Klebsiella pneumoniae, Moraxella catarrhalis, Mycoplasma pneumoniae, Chlamydia pneumoniae, Haemophilus influenzae*; withdrawn by the EMA in 2009 due to concern for genotoxic side effects. | [3,7,12–14] |
| Besifloxacin (4th) | Topical use (ophthalmic); bacterial conjunctivitis. | [15,16] |
| Finafloxacin (4th) | Acute otitis externa caused by susceptible strains of *Pseudomonas aeruginosa* and *Staphylococcus aureus*. | [17,18] |
| Delafloxacin (4th) | Acute bacterial skin and skin structure infections caused by *Staphylococcus aureus* (including MRSA), *Staphylococcus haemolyticus, Staphylococcus lugdunensis, Streptococcus pyogenes, Streptococcus agalactiae, Escherichia coli, Klebsiella pneumoniae, Enterobacter cloacae, Pseudomonas aeruginosa;*<br>Community-acquired bacterial pneumonia caused by *Streptococcus pneumoniae, Staphylococcus aureus* (methicillin-susceptible (MSSA) isolates only), *Klebsiella pneumoniae, Escherichia coli, Pseudomonas aeruginosa, Haemophilus influenzae, Haemophilus parainfluenzae, Chlamydia pneumoniae, Legionella pneumophila, Mycoplasma pneumoniae.* | [19,20] |

**Table S2.** Side effects/adverse reactions of the antibacterial (F)QNs [1].

| Affected system | Symptom | Higher risk | Lower risk |
|---|---|---|---|
| **Musculoskeletal and connective tissues** | Arthralgia | Ciprofloxacin | Norfloxacin, Ofloxacin (1-3%), Levofloxacin (0.1-1%), Moxifloxacin (0.1-1%), Gemifloxacin (≤0.1%) |
| | Back pain | | Moxifloxacin (0.1-1%), Gemifloxacin (≤0.1%) |
| | Joint stiffness | | Ciprofloxacin |
| | Leg cramps | | Gemifloxacin (≤0.1%) |
| | Muscle injuries (including rupture) | | Levofloxacin (postmarketing) |
| | Muscle spasms | | Norfloxacin, Moxifloxacin (0.1-1%) |
| | Muscle weakness | | Ciprofloxacin, Moxifloxacin (postmarketing) |
| | Myalgia | Ciprofloxacin, Delafloxacin (<2%) | Norfloxacin, Ofloxacin (1-3%), Levofloxacin (0.1-1%) |
| | Myastenia | | Ciprofloxacin |
| | *Myastenia Gravis* (exacerbation) | | Ciprofloxacin, Norfloxacin, Moxifloxacin (postmarketing) |
| | Pain in extremity | | Moxifloxacin (0.1-1%) |
| | Rhabdomyolysis | | Levofloxacin (postmarketing) |
| | Skeletal (and/or Musculoskeletal) pain | | Levofloxacin (0.1-1%), Moxifloxacin (0.1-1%) |
| | Tendinitis | Ciprofloxacin, Norfloxacin | Nalidixic acid, Levofloxacin (0.1-1%) |
| | Tendon ruptures (including Achilles tendon) | Ciprofloxacin, Norfloxacin | Nalidixic acid, Levofloxacin (postmarketing), Moxifloxacin (postmarketing) |
| **Central nervous system and psychiatric disorders** | 6th cranial nerve palsy | | Nalidixic acid |
| | Abnormal EEG (electroencephalogram) | | Levofloxacin (postmarketing) |
| | Abnormal gait | | Ciprofloxacin, Moxifloxacin (postmarketing) |
| | Agitation | Ciprofloxacin | Pefloxacin, Levofloxacin (0.1-1%), Moxifloxacin (0.1-1%) |
| | Altered coordination | | Moxifloxacin (postmarketing) |
| | Anorexia | | Ciprofloxacin, Norfloxacin (0.3-1.0%), Levofloxacin (0.1-1%), Moxifloxacin (0.1-1%), Gemifloxacin (0.1-1%) |
| | Anxiety | Ciprofloxacin, Delafloxacin (<2%) | Pefloxacin, Ofloxacin (1-3%), Levofloxacin (0.1-1%), Moxifloxacin (0.1-1%) |

| Affected system | Symptom | Higher risk | Lower risk |
|---|---|---|---|
| | Asthenia | | Norfloxacin (0.3-1.0%), Ofloxacin (<1%), Moxifloxacin (0.1-1%), Gemifloxacin (≤0.1%) |
| | Ataxia | | Ciprofloxacin, Norfloxacin |
| | Brief convulsions/ Seizures (eg. *Status Epilepticus*) | Ciprofloxacin | Nalidixic acid, Norfloxacin, Ofloxacin (1-3%), Levofloxacin (0.1-1%) |
| | Cognitive change | | Ofloxacin (1-3%) |
| | Confusional state | Ciprofloxacin | Norfloxacin, Ofloxacin (1-3%), Levofloxacin (0.1-1%), Moxifloxacin (0.1-1%) |
| | Delirium | | Ciprofloxacin, |
| | Depersonalisation | | Ciprofloxacin |
| | Depression | Ciprofloxacin | Ofloxacin (1-3%), Levofloxacin (0.1-1%), Moxifloxacin (0.1-1%) |
| | Disorientation | | Moxifloxacin (0.1-1%) |
| | Dizziness | Nalidixic acid, Ciprofloxacin, Norfloxacin (2.6%), Levofloxacin (≥1), Moxifloxacin (≥1%), Gemifloxacin (1.7%), Delafloxacin (<2%) | |
| | Dream abnormality | Delafloxacin (<2%) | Ofloxacin (1-3%), Levofloxacin (0.1-1%) |
| | Drowsiness | Nalidixic acid | |
| | Encephalopathy | | Levofloxacin (postmarketing) |
| | Euphoria | | Ofloxacin (1-3%) |
| | Guillain-Barré | | Norfloxacin |
| | Headache | Nalidixic acid, Ciprofloxacin, Norfloxacin (2.0%), Ofloxacin (9%), Levofloxacin (≥1), Moxifloxacin (≥1%), Gemifloxacin (4.2%), Besifloxacin (1-2%), Delafloxacin (3%), Finafloxacin (1.8%) | |
| | Hallucinations | Ciprofloxacin | Norfloxacin, Ofloxacin (1-3%), Levofloxacin (0.1-1%), Moxifloxacin (0.1-1%) |
| | Hyperkinesias | | Levofloxacin (0.1-1%) |
| | Hypertonia | | Ciprofloxacin, Levofloxacin (0.1-1%) |
| | Increased intracranial pressure | Ciprofloxacin | Nalidixic acid, Norfloxacin |
| | Insomnia | Ciprofloxacin, Ofloxacin (7%), Levofloxacin (≥1), Moxifloxacin (≥1%), Delafloxacin (<2%) | Gemifloxacin (0.1-1%) |
| | Irritability | | Ciprofloxacin |
| | Lethargy | | Moxifloxacin (0.1-1%) |
| | Malaise | | Ciprofloxacin, Ofloxacin (<1%), Moxifloxacin (0.1-1.%) |

| Affected system | Symptom | Higher risk | Lower risk |
|---|---|---|---|
| | Manic reaction | | Ciprofloxacin |
| | Migraine | | Ciprofloxacin |
| | Myoclonus | | Ciprofloxacin, Norfloxacin |
| | Nervousness | Ciprofloxacin, Ofloxacin (1-3%) | Pefloxacin, Moxifloxacin (0.1-1%) |
| | Nightmares | Ciprofloxacin | Levofloxacin (0.1-1%) |
| | Paranoia | Ciprofloxacin | Levofloxacin (postmarketing) |
| | Phobia | | Ciprofloxacin |
| | Psychotic reactions | | Norfloxacin, Levofloxacin (postmarketing), Moxifloxacin (postmarketing) |
| | Restlessness | | Ciprofloxacin, Norfloxacin, Moxifloxacin (0.1-1%) |
| | Sleep disorders | Ofloxacin (1-3%) | Levofloxacin (0.1-1%) |
| | Somnolence | Ofloxacin (1-3%) | Levofloxacin (0.1-1%), Moxifloxacin (0.1-1%), Gemifloxacin (0.1-1%) |
| | Suicide attempt and Suicidal ideation | | Levofloxacin (postmarketing), Moxifloxacin (postmarketing) |
| | (Pre)Syncope | Ofloxacin (1-3%), Delafloxacin (<2%) | Levofloxacin (0.1-1%), Moxifloxacin (0.1-1%), Gemifloxacin (postmarketing) |
| | Toxic psychosis | Ciprofloxacin | Nalidixic acid, Norfloxacin |
| | Transient ischemic attack | | Gemifloxacin (postmarketing) |
| | Tremor | Ciprofloxacin | Norfloxacin, Ofloxacin (1-3%), Levofloxacin (0.1-1%), Moxifloxacin (0.1-1%), Gemifloxacin (≤0.1%) |
| | Twitching | | Ciprofloxacin |
| | Vertigo | Nalidixic acid | Ofloxacin (1-3%), Levofloxacin (0.1-1%), Gemifloxacin (≤0.1%) |
| | Weakness | Nalidixic acid, Ciprofloxacin | Norfloxacin |
| **Peripheral nervous system** | Dyesthesia | Ciprofloxacin | Nalidixic acid, Norfloxacin |
| | Hypoesthesia | Ciprofloxacin, Delafloxacin (<2%) | Nalidixic acid, Norfloxacin, Moxifloxacin (0.1-1%) |
| | Paresthesia | Ciprofloxacin, Delafloxacin (<2%) | Nalidixic acid, Norfloxacin (0.3-1.0%), Ofloxacin (1-3%), Levofloxacin (0.1-1%), Moxifloxacin (0.1-1%) |
| | Peripheral neuropathy | Ciprofloxacin | Nalidixic acid, Pefloxacin, Norfloxacin, Levofloxacin (postmarketing), Moxifloxacin (postmarketing) |
| | Polyneuropathy | | Ciprofloxacin, Moxifloxacin (postmarketing) |
| **Eyes/ Ears/ Nose/ Mouth** | Ageusia | | Ciprofloxacin, Levofloxacin (postmarketing) |
| | Anosmia | | Ciprofloxacin, Levofloxacin (postmarketing) |
| | Blurred vision | Besifloxacin (1-2%), Delafloxacin (<2%) | Ciprofloxacin, Levofloxacin (postmarketing), Moxifloxacin (0.1-1%) |

| Affected system | Symptom | Higher risk | Lower risk |
|---|---|---|---|
| | Conjuctival redness | Besifloxacin (2%) | |
| | Decreased visual acuity | | Ciprofloxacin, Levofloxacin (postmarketing) |
| | Diplopia | | Ciprofloxacin, Norfloxacin, Levofloxacin (postmarketing) |
| | Disturbed vision (eg. chromatopsia and photopsia) | Ofloxacin (1-3%) | Ciprofloxacin, Levofloxacin (postmarketing), Gemifloxacin (≤0.1%) |
| | Dry mouth | Ofloxacin (1-3%) | Moxifloxacin (0.1-1%), Gemifloxacin (0.1-1%) |
| | Dysgeusia | Delafloxacin (<2%), Ofloxacin (1-3%) | Ciprofloxacin, Norfloxacin, Levofloxacin (postmarketing), Moxifloxacin (0.1-1%), Gemifloxacin (0.1-1%) |
| | Dysphonia | | Levofloxacin (postmarketing) |
| | Ear discomfort | | Finafloxacin (0.3%) |
| | Ear pain | | Finafloxacin (0.5%) |
| | Ear pruritus | Finafloxacin (1.3%) | |
| | Epistaxis | | Levofloxacin (0.1-1%), Ofloxacin (<1%) |
| | Eye irritation | Besifloxacin (1-2%) | |
| | Eye pain | Besifloxacin (1-2%) | |
| | Eye pruritus | Besifloxacin (1-2%) | |
| | Glossitis | | Levofloxacin (0.1-1%), |
| | Hearing loss | | Ciprofloxacin, Norfloxacin, Ofloxacin (1-3%), Levofloxacin (postmarketing), Moxifloxacin (postmarketing) |
| | Nystagmus | | Ciprofloxacin, Norfloxacin |
| | Oral candidiasis | Delafloxacin (<2%) | Ciprofloxacin |
| | Otitis externa | Finafloxacin (1.8%) | |
| | Otitis media | Finafloxacin (1.3%) | |
| | Papilledema | | Nalidixic acid |
| | Parosmia | | Levofloxacin (postmarketing) |
| | Photophobia | | Ofloxacin (1-3%) |
| | Retinal hemorrhage | | Gemifloxacin (postmarketing) |
| | Scotoma | | Levofloxacin (postmarketing) |
| | Stomatitis | | Norfloxacin, Levofloxacin (0.1-1%), |
| | Tinnitus | Delafloxacin (<2%) | Ciprofloxacin, Norfloxacin, Ofloxacin (1-3%), Levofloxacin (postmarketing), Moxifloxacin (0.1-1%) |
| | Vertigo | Delafloxacin (<2%) | Moxifloxacin (0.1-1%) |
| | Vision loss | | Moxifloxacin (postmarketing) |
| | Uveitis | | Norfloxacin |
| **Cardiovascular system** | *Angina Pectoris* | | Ciprofloxacin, Moxifloxacin (0.1-1%) |
| | Arrhythmia | | Ciprofloxacin, Norfloxacin |
| | Atrial fibrillation | | Moxifloxacin (0.1-1%) |
| | Bradycardia | Delafloxacin (<2%) | Moxifloxacin (0.1-1%) |

| Affected system | Symptom | Higher risk | Lower risk |
|---|---|---|---|
| | Caridiac failure | | Moxifloxacin (0.1-1%) |
| | Cardiopulmonary arrest | | Ciprofloxacin, Ofloxacin (<1%), Levofloxacin (0.1-1%), , Moxifloxacin (0.1-1%), Moxifloxacin (postmarketing) |
| | Cardiovascular collapse | | Norfloxacin |
| | Edema | Levofloxacin (≥1%), Delafloxacin (<2%) | Ofloxacin (<1%), Moxifloxacin (0.1-1%), Gemifloxacin (postmarketing) |
| | Hypertension | Delafloxacin (<2%) | Ofloxacin (<1%), Moxifloxacin (0.1-1%) |
| | Hypotension | Delafloxacin (<2%) | Ciprofloxacin, Norfloxacin, Ofloxacin (<1%), Moxifloxacin (0.1-1%) |
| | Myocardial infarction | | Ciprofloxacin |
| | Palpitations | Delafloxacin (<2%) | Ofloxacin (<1%), Levofloxacin (0.1-1%), Moxifloxacin (0.1-1%) |
| | Supraventricular tachycardia | | Gemifloxacin (postmarketing) |
| | Syncope | | Ciprofloxacin, Norfloxacin, |
| | Sinus tachycardia | Delafloxacin (<2%) | Ciprofloxacin, Levofloxacin (postmarketing), Moxifloxacin (0.1-1%) |
| | *Torsade de pointes* | | Ciprofloxacin, Norfloxacin, Levofloxacin (postmarketing), Moxifloxacin (postmarketing) |
| | Vasodilation | | Ofloxacin (<1%), Levofloxacin (postmarketing) |
| | Ventricular arrythmia | | Ciprofloxacin, Levofloxacin (0.1-1%), |
| | Ventricular tachyarrhythmias | | Moxifloxacin (postmarketing) |
| | Ventricular tachycardia | | Levofloxacin (0.1-1%), |
| | QTc interval prolongation | | Nalidixic acid, Ciprofloxacin, Norfloxacin, Levofloxacin (postmarketing), Moxifloxacin (0.1-1%), Gemifloxacin (postmarketing) |
| **Respiratory tract** | Asthma | | Moxifloxacin (0.1-1%) |
| | Bronchospasm | | Ciprofloxacin, Moxifloxacin (0.1-1%) |
| | Cough | | Ofloxacin (1-3%) |
| | Dyspnea | Levofloxacin (≥1%) | Ciprofloxacin, Norfloxacin, Moxifloxacin (0.1-1%), Gemifloxacin (≤0.1%) |
| | Hemoptysis | | Ciprofloxacin |
| | Laryngeal edema | | Ciprofloxacin |
| | Pharyngeal edema | | Ciprofloxacin, Norfloxacin |

| Affected system | Symptom | Higher risk | Lower risk |
|---|---|---|---|
| | Pharyngitis | Ofloxacin (1-3%) | Gemifloxacin (≤0.1%) |
| | Pneumonia | | Gemifloxacin (≤0.1%) |
| | Respiratory arrest | | Ofloxacin (1-3%) |
| | Rhinorrhea | | Ofloxacin (1-3%) |
| | Wheezing | | Moxifloxacin (0.1-1%) |
| **Gastrointestinal tract** | Abdominal disconfort/pain | Nalidixic acid, Norfloxacin (1.6%), Ofloxacin (1-3%), Levofloxacin (≥1%), Moxifloxacin (≥1%), Gemifloxacin (2.2%), Delafloxacin (<2%) | Ciprofloxacin |
| | Abdominal distension | | Moxifloxacin (0.1-1%) |
| | Constipation | Ofloxacin (1-3%), Levofloxacin (≥1%), Moxifloxacin (≥1%) | Norfloxacin (0.3-1.0%), Gemifloxacin (0.1-1%) |
| | Diarrhea | Nalidixic acid, Ciprofloxacin (1.6%), Ofloxacin (4%), Levofloxacin (≥1%), Moxifloxacin (≥1%), Gemifloxacin (5.0%), Delafloxacin (8%) | Norfloxacin (0.3-1.0%) |
| | Dyspepsia | Levofloxacin (≥1%), Moxifloxacin (≥1%), Delafloxacin (<2%) | Norfloxacin (0.3-1.0%), Ofloxacin (<1%), Gemifloxacin (0.1-1%) |
| | Dysphagia | | Norfloxacin |
| | Esophagitis | | Levofloxacin (0.1-1%), |
| | Flatulence | Ofloxacin (1-3%) | Norfloxacin (0.3-1.0%), Moxifloxacin (0.1-1%), Gemifloxacin (0.1-1%) |
| | Gastritis | | Levofloxacin (0.1-1%), Moxifloxacin (0.1-1%), Gemifloxacin (0.1-1%) |
| | Gastroenteritis | | Levofloxacin (0.1-1%), Moxifloxacin (0.1-1%), Gemifloxacin (≤0.1%) |
| | Gastrointestinal bleeding | | Ciprofloxacin |
| | Gastrointestinal candidias | | Ciprofloxacin |
| | Gastrointestinal distress | Ofloxacin (1-3%) | |
| | Gastroesophageal reflux disease | | Moxifloxacin (0.1-1%) |
| | Intestinal perforation | | Ciprofloxacin |
| | Nausea | Nalidixic acid, Ciprofloxacin (2.5%), Norfloxacin (2.6%), Ofloxacin (10%), Levofloxacin (≥1%), Moxifloxacin (≥1%), Gemifloxacin (3.7%), Delafloxacin (8%), Finafloxacin (1%) | |

| Affected system | Symptom | Higher risk | Lower risk |
|---|---|---|---|
| | Non-specified gatrointestinal disorder | | Gemifloxacin (≤0.1%) |
| | Pancreatitis | | Ciprofloxacin, Norfloxacin, Levofloxacin (0.1-1%), |
| | Pseudomembranous colitis (eg. *Clostridium difficile*) | Delafloxacin (<2%) | Ciprofloxacin, Norfloxacin, Levofloxacin (0.1-1%), Gemifloxacin (postmarketing - antibiotic-associated colitis) |
| | Vomiting | Nalidixic acid, Ciprofloxacin (1%), Ofloxacin (4%), Levofloxacin (≥1%), Moxifloxacin (≥1%), Gemifloxacin (1.6%), Delafloxacin (2%) | Norfloxacin (0.3-1.0%), Gemifloxacin (0.1-1%) |
| **Hepatobiliary Disorders** | Abnormal hepatic function | Ciprofloxacin (1.3%) | Levofloxacin (0.1-1%), Moxifloxacin (0.1-1.%) |
| | Acute hepatic failure | | Ciprofloxacin, Norfloxacin, Levofloxacin (postmarketing), Moxifloxacin (postmarketing) |
| | Acute hepatic necrosis | | Ciprofloxacin, Norfloxacin, Moxifloxacin (postmarketing) |
| | Cholestasis/ Cholestatic jaundice | | Nalidixic acid, Ciprofloxacin, Norfloxacin, Levofloxacin (postmarketing), Moxifloxacin (postmarketing) |
| | Hepatitis | | Ciprofloxacin, Norfloxacin, Levofloxacin (postmarketing), Moxifloxacin (postmarketing) |
| **Renal and Urinary Disorders** | Abnormal renal function | Delafloxacin (<2%) | Levofloxacin (0.1-1%) |
| | Abnormal urine | | Gemifloxacin (≤0.1%) |
| | Acute renal insufficiency or failure | Delafloxacin (<2%) | Ciprofloxacin, Norfloxacin, Levofloxacin (0.1-1%), Moxifloxacin (0.1-1%), Gemifloxacin (postmarketing) |
| | Dysuria | | Ofloxacin (1-3%) Moxifloxacin (0.1-1%) |
| | Interstitial nephritis | | Ciprofloxacin, Norfloxacin, Levofloxacin (postmarketing), Moxifloxacin (postmarketing) |
| | Urinary retention | | Ofloxacin (1-3%) |
| | Crystalluria | | Ciprofloxacin, Norfloxacin |
| | Albuminuria | | Norfloxacin |
| | Candiduria | | Norfloxacin |
| | Cylindruria | | Ciprofloxacin, Norfloxacin |
| | Hematuria | | Ciprofloxacin, Norfloxacin |
| | Agranulocytosis | | Ciprofloxacin, Norfloxacin, Moxifloxacin (postmarketing) |

| Affected system | Symptom | Higher risk | Lower risk |
|---|---|---|---|
| **Blood/ Lymphatic system** | Anaemia (eg. hemolytic, aplastic) | Moxifloxacin (≥1%) | Nalidixic acid, Ciprofloxacin, Norfloxacin, Levofloxacin (0.1-1%), Gemifloxacin (≤0.1%) |
| | Granulocytopenia | | Levofloxacin (0.1-1%), Gemifloxacin (≤0.1%) |
| | Leukocytosis | | Ciprofloxacin, Moxifloxacin (0.1-1%) |
| | Leukopenia | | Nalidixic acid, Ciprofloxacin, Norfloxacin, Levofloxacin (postmarketing), Moxifloxacin (0.1-1%), Gemifloxacin (0.1-1%) |
| | Monocytosis | | Ciprofloxacin |
| | Neutropenia | | Norfloxacin, Moxifloxacin (0.1-1%), Gemifloxacin (0.5%) |
| | Neutrophilia | | Gemifloxacin (0.5%) |
| | Pancytopenia | | Ciprofloxacin, Norfloxacin, Levofloxacin (postmarketing), Moxifloxacin (postmarketing) |
| | Petechia | | Ciprofloxacin |
| | Thrombocythemia | | Ciprofloxacin, Moxifloxacin (0.1-1%), Gemifloxacin (0.1-1%) |
| | Thrombocytopenia | | Nalidixic acid, Ciprofloxacin, Norfloxacin, Levofloxacin (0.1-1%), Moxifloxacin (0.1-1%), Gemifloxacin (≤0.1%) |
| | Thrombotic thrombocytopenic purpura | | Ciprofloxacin, Norfloxacin |
| **Metabolism and Nutrition Disorders** | Cholesterol elevation | | |
| | Decreased appetite | Ofloxacin (1-3%) | Moxifloxacin (0.1-1%) |
| | Dehydration | | Moxifloxacin (0.1-1%) |
| | Hyperglycemia | Delafloxacin (<2%) | Ciprofloxacin, Levofloxacin (0.1-1%), Moxifloxacin (0.1-1%), Gemifloxacin (0.1-1%) |
| | Hyperlipidemia | | Moxifloxacin (0.1-1%) |
| | Hypoglycemia | Delafloxacin (<2%) | Ciprofloxacin, Norfloxacin, Levofloxacin (0.1-1%) |
| | Thirst | | Ofloxacin (1-3%) |
| | Weight Loss | | Ofloxacin (1-3%) |
| **Skin and Allergic Reactions** | Acute generalised exanthematous pustulosis | | Ciprofloxacin |
| | Allergic dermatitis | | Moxifloxacin (0.1-1%), Gemifloxacin (0.1-1%) |
| | Allergic pneumonitis | | Ciprofloxacin, Norfloxacin, Levofloxacin (postmarketing), Moxifloxacin (postmarketing) |
| | Allergic reaction | | Levofloxacin (0.1-1%) |

| Affected system | Symptom | Higher risk | Lower risk |
|---|---|---|---|
| | Anaphylactoid reaction (including anaphylactic shock) | | Nalidixic acid, Ciprofloxacin, Norfloxacin, Levofloxacin (postmarketing), Moxifloxacin (postmarketing), Gemifloxacin (postmarketing) |
| | Angioedema (including laryngeal edema) | | Nalidixic acid, Ciprofloxacin, Norfloxacin, Ofloxacin (1-3%), Moxifloxacin (postmarketing) |
| | Angioneurotic edema | | Levofloxacin (postmarketing) |
| | Arthralgia with joint stiffness and swelling | | Nalidixic acid |
| | Atopic dermatitis | | Gemifloxacin (≤0.1%) |
| | Contact dermatitis | | Nadifloxacin |
| | Dermatitis | Delafloxacin (<2%) | |
| | Diaphoresis | | Ofloxacin (1-3%) |
| | Dry skin | | Nadifloxacin |
| | Erythema | Delafloxacin (<2%) | Nadifloxacin, Moxifloxacin (0.1-1%) |
| | *Erythema Multiforme* | | Nalidixic acid, Ciprofloxacin, Levofloxacin (postmarketing), Gemifloxacin (postmarketing) |
| | *Erythema Nodosum* | | Ciprofloxacin |
| | Exfoliative dermatitis | | Ciprofloxacin |
| | Fever | Ofloxacin (1-3%), Moxifloxacin (≥1%) | Ciprofloxacin, Norfloxacin, Levofloxacin (postmarketing) |
| | Fixed eruption | | Ciprofloxacin |
| | Flushing | Delafloxacin (<2%) | Ciprofloxacin, Gemifloxacin (≤0.1%), Nadifloxacin |
| | Hypersensitivity | Delafloxacin (<2%) | Ciprofloxacin |
| | Hypopigmentation of the skin | | Nadifloxacin |
| | Leukocytoclastic vasculitis | | Levofloxacin (postmarketing) |
| | Papules | | Nadifloxacin |
| | Photosensitivity/ Phototoxicity reactions | | Nalidixic acid, Ciprofloxacin, Norfloxacin, Levofloxacin (postmarketing), Moxifloxacin (postmarketing), Gemifloxacin (0.039%) |
| | Pruritus | Nalidixic acid, Ofloxacin (1-3%), Nadifloxacin (>1.8%), Levofloxacin (≥1%), Delafloxacin (<2%) | Ciprofloxacin, Pefloxacin, Norfloxacin, Moxifloxacin (0.1-1%), Gemifloxacin (0.1-1%) |
| | Rash | Nalidixic acid, Ciprofloxacin (1%), Norfloxacin, Ofloxacin (1-3%), Levofloxacin (≥1%), Gemifloxacin (3.5%), Delafloxacin (<2%) | Pefloxacin, Moxifloxacin (0.1-1%) |

| Affected system | Symptom | Higher risk | Lower risk |
|---|---|---|---|
| | Serum sickness | | Ciprofloxacin, Norfloxacin, Levofloxacin (postmarketing) |
| | Skin exfoliation | | Gemifloxacin (postmarketing) |
| | Skin irritation | Delafloxacin (<2%) | Nadifloxacin |
| | Skin warmth | | Nadifloxacin |
| | Stevens-Johnson Syndrome | | Nalidixic acid, Ciprofloxacin, Norfloxacin, Levofloxacin (postmarketing), Moxifloxacin (postmarketing) |
| | Sweating | | Ciprofloxacin |
| | Toxic epidermal necrolysis | | Levofloxacin (postmarketing), Moxifloxacin (postmarketing) |
| | Urticaria | Nalidixic acid, Delafloxacin (<2%) | Ciprofloxacin, Pefloxacin, Norfloxacin, Ofloxacin (1-3%), Nadifloxacin, Levofloxacin (0.1-1%), Moxifloxacin (0.1-1%), Gemifloxacin (0.1-1%) |
| **Genital/ Reproductive System** | Burning, pain and rash of the female genitalia | | Ofloxacin (<1%) |
| | Dysmenorrhea | | Ofloxacin (<1%) |
| | External genital pruritus (in women) | Ofloxacin (6%) | Gemifloxacin (0.1-1%) |
| | Genital moniliasis | | Levofloxacin (0.1-1%), Gemifloxacin (0.1-1%) |
| | Menorrhagia | | Ofloxacin (<1%) |
| | Metrorrhagia | | Ofloxacin (<1%) |
| | Vaginal and/ or Vulvovaginal candidiasis | Delafloxacin (<2%) | Ciprofloxacin, Norfloxacin, Moxifloxacin (0.1-1.%) |
| | Vaginal discharge | Ofloxacin (1-3%) | |
| | Vaginal infection | | Moxifloxacin (0.1-1.%) |
| | Vaginitis | Ofloxacin (5%), Levofloxacin (≥1%) | Gemifloxacin (0.1-1%) |
| **Laboratory Abnormalities** | Decreased CPK (Creatine Phosphokinase) | | Gemifloxacin (0.2%) |
| | Decreased Hematocrit and/or Hemoglobin | | Norfloxacin (0.6%), Moxifloxacin (0.1-1%), Gemifloxacin (0.3%) |
| | Decreased Platelet Count | Norfloxacin(1.0%) | Gemifloxacin (0.2%) |
| | Decreased PT (Prothrombin Time) | | Ciprofloxacin |
| | Decreased RBC (Red Blood Cells) | | Gemifloxacin (0.1%) |
| | Decreased Total Protein | | Gemifloxacin (0.2%) |

| Affected system | Symptom | Higher risk | Lower risk |
|---|---|---|---|
| | Decreased WBC (White Blood Cells) | Norfloxacin (1.3%) | |
| | Eosinophilia | | Ciprofloxacin, Levofloxacin (postmarketing), Norfloxacin (0.6%), Moxifloxacin (0.1-1%), Naladixic acid |
| | Hypercalcemia | | Gemifloxacin (<0.1%) |
| | Hyperkalemia | | Ciprofloxacin, Levofloxacin (0.1-1%), Gemifloxacin (0.3%) |
| | Hypernatremia | | Gemifloxacin (0.1%) |
| | Hypoalbuminemia | | Gemifloxacin (0.3%) |
| | Hypocalcemia | | Gemifloxacin (0.1%) |
| | Hypokalemia | Moxifloxacin (≥1%) | Gemifloxacin (0.1%) |
| | Hyponatremia | | Gemifloxacin (0.1-1%) |
| | Increased ALP (Alkaline Phosphatase) | Delafloxacin (<2%) | Ciprofloxacin, Levofloxacin (0.1-1%), Moxifloxacin (0.1-1%), Gemifloxacin (0.4%) |
| | Increased ALT (Alanine Transaminase) | Moxifloxacin (≥1%) Gemifloxacin (1.7%) | Ciprofloxacin |
| | Increased AST (Aspartate Aminotransferase) (SGOT - Serum glutamic oxaloacetic transaminase) | Norfloxacin (1.6%), Gemifloxacin (01.3%) | Ciprofloxacin, Moxifloxacin (0.1-1%) |
| | Increased blood amylase | | Moxifloxacin (0.1-1%) |
| | Increased blood CPK (Creatine Phosphokinase) | Delafloxacin (<2%) | Ciprofloxacin, Moxifloxacin (0.1-1%), Gemifloxacin (0.7%) |
| | Increased blood uric acid | | Ciprofloxacin, Moxifloxacin (0.1-1%) |
| | Increased BUN (Blood Urea Nitrogen) | | Ciprofloxacin, Moxifloxacin (0.1-1%), Gemifloxacin (0.3%) |
| | Increased LDH (Lactate Dehydrogenase) | | Ciprofloxacin, Moxifloxacin (0.1-1%), Gemifloxacin (<0.1%) |
| | Increased lipase | | Norfloxacin (0.6%) |
| | Increased GGT (gamma-Glutamyl Transferase) | | Moxifloxacin (0.1-1%), Gemifloxacin (≤0.1%) |
| | Increased Hematocrit and/or Hemoglobin | | Ciprofloxacin, Gemifloxacin (0.1+ %) |
| | Increased hepatic enzymes | Delafloxacin (3%) | Levofloxacin (0.1-1%), |

| Affected system | Symptom | Higher risk | Lower risk |
|---|---|---|---|
| | Increased muscle enzymes | | Levofloxacin (postmarketing) |
| | Increased non-proteins Nitrogen | | Gemifloxacin (≤0.1%) |
| | Increased triglycerides | | Moxifloxacin (0.1-1%) |
| | Methemoglobinemia | | Ciprofloxacin |
| | Prolonged aPTT (activated Partial Thromboplastin Time) | | Moxifloxacin (0.1-1%) |
| | Prolonged INR (International Normalized Ratio) | | Ciprofloxacin, Gemifloxacin (postmarketing) |
| | Prolonged PT (Prothrombin Time) | | Ciprofloxacin, Levofloxacin (postmarketing), Moxifloxacin (0.1-1%) |
| **Other** | Anal/ Rectal pain | | Norfloxacin (0.3-1.0%) |
| | Bleeding diathesis | | Ciprofloxacin |
| | Chest discomfort/ pain | Ofloxacin (1-3%), Levofloxacin (≥1%) | Moxifloxacin (0.1-1.%), Moxifloxacin (0.1-1.%) |
| | Chills | | Ofloxacin (<1%), Moxifloxacin (0.1-1.%) |
| | Discomfort | Delafloxacin (<2%) | |
| | Extremity pain | | Ofloxacin (<1%) |
| | Facial edema | | Ciprofloxacin, Gemifloxacin (≤0.1%) |
| | Facial pain | | Moxifloxacin (0.1-1.%) |
| | Fatigue | Ofloxacin (1-3%) | Moxifloxacin (0.1-1%), Gemifloxacin (0.1-1%) |
| | Fungal infections | Delafloxacin (<2%) | Moxifloxacin (0.1-1%), Gemifloxacin (0.1-1%) |
| | Hemorrhage | | Gemifloxacin (postmarketing) |
| | Hot flashes | | Gemifloxacin (≤0.1%) |
| | Hyperhidrosis | | Norfloxacin (0.3-1.0%), Moxifloxacin (0.1-1%) |
| | Infusion site bruise | Delafloxacin (<2%) | |
| | Injection site reactions | Levofloxacin (≥1%), Delafloxacin (<2%) | Moxifloxacin (0.1-1.%) |
| | Metabolic acidosis | | Nalidixic acid, Ciprofloxacin, Norfloxacin |
| | Monoliasis | Levofloxacin (≥1) | |
| | Multiple organ dysfunction syndrome | | Levofloxacin (postmarketing) |
| | Night Sweats | | Moxifloxacin (0.1-1%) |

| Affected system | Symptom | Higher risk | Lower risk |
|---|---|---|---|
| | Pain | | Ciprofloxacin, Ofloxacin (<1%), Moxifloxacin (0.1-1.%), Gemifloxacin (≤0.1%) |
| | Phlebitis | Delafloxacin (<2%) | Levofloxacin (0.1-1%), Moxifloxacin (0.1-1%) |
| | Swelling | Delafloxacin (<2%) | |
| | Thrombosis | Delafloxacin (<2%) | |
| | Trunk pain | Ofloxacin (1-3%), Delafloxacin (<2%) | |
| | Vasculitis | | Ciprofloxacin, Norfloxacin, Ofloxacin (1-3%) |

[1] References: Besifloxacin [21], Ciprofloxacin [22], Delafloxacin [23], Finafloxacin [18,24], Gemifloxacin [25], Levofloxacin [26], Moxifloxacin [27], Nadifloxacin [28], Nalidixic acid [29], Norfloxacin [30], Ofloxacin [31], Pefloxacin [32].

**Table S3.** Pathogens classified by WHO according to the urgency of antibiotic discovery need [33].

| Critical priority | High priority | Medium priority |
|---|---|---|
| *Acinetobacter baumannii*, carbapenem – R [1] | *Enterococcus faecium*, vancomycin – R | *Streptococcus pneumoniae*, penicillin-non-susceptible |
| *Pseudomonas aeruginosa*, carbapenem – R | *Staphylococcus aureus*, methicillin – R vancomycin – I [1], R | *Haemophilus influenzae*, ampicillin – R |
| Enterobacteriaceae, carbapenem – R ESBL [1]-producing | *Helicobacter pylori*, clarithromycin – R | *Shigella* spp., fluoroquinolone – R |
| | *Campylobacter* spp., fluoroquinolone – R | |
| | *Salmonellae*, fluoroquinolone – R | |
| | *Neisseria gonorrhoeae*, cephalosporin – R fluoroquinolone – R | |

[1] R = resistant; ESBL = extended spectrum β-lactamase; I = intermediate

**Table S4.** The emergence of resistance to newer antibiotics (Ref. = references).

| Antibiotic | Resistant bacteria | Resistance rate | Ref. |
| --- | --- | --- | --- |
| Tigecycline (initial US Approval: 2005) [34] | *Acinetobacter baumannii* | – | [35] |
| Ceftolozane/tazobactam (initial US Approval: 2014) [36] | *Pseudomonas aeruginosa* | 0 – 8.3% | [37] |
| Ceftazidime/avibactam (initial US Approval: 2015) [38] | Enterobacteriaceae | <0.6% (overall) | [39–43] |
| | | <2.3 % (MDR [1] and XDR [1] strains) | [40,44,45] |
| | | 24.7% (CR [1] strains) | [41,46,47] |
| | | 90.8 – 98.6% (most MBL [1]-positive strains) | [41,42,48] |
| | *Klebsiella pneumoniae* | <5.2% (overall) | [48–50] |
| | | 16.7% or up to 21% (CR [1] strains) | [50,51] |
| | *Pseudomonas aeruginosa* | 2.9% | [44] |
| | | 18% | [52] |
| | | >95% (MBL [1]-positive strains) | [48,53] |
| | *Acinetobacter baumannii* | >55% | [54,55] |
| | | 73.6% (strains from intensive care units) | [54] |
| Cefiderocol (initial US Approval: 2019) [56] | *Acinetobacter baumannii* | – | [57] |

[1] MDR = multidrug-resistant; XDR = extensively drug-resistant; CR = carbapenem-resistant; MBL = metallo-β-lactamase; ESBL = extended spectrum β-lactamase

**References**


1. *Wilson and Gisvold's Textbook of Organic Medicinal and Pharmaceutical Chemistry*; Wilson, C.O., Beale, J.M., Block, JH, Eds.; 12th ed.; Lippincott Williams & Wilkins: Baltimore, MD, 2011; ISBN 978-0-7817-7929-6.
2. Gonzalez, J.P.; Henwood, J.M. Pefloxacin. A Review of Its Antibacterial Activity, Pharmacokinetic Properties and Therapeutic Use. *Drugs* **1989**, *37*, 628–668, doi:10.2165/00003495-198937050-00003.
3. *Martindale: The Complete Drug Reference*; Martindale, W., Sweetman, S.C., Eds.; 36. ed.; Pharmaceuticale Press, PhP: London ; Chicago, 2009; ISBN 978-0-85369-840-1.
4. Nenoff, P. Acne Vulgaris and Bacterial Skin Infections: Review of the Topical Quinolone Nadifloxacin. *Expert Review of Dermatology* **2006**, *1*, 643–654, doi:10.1586/17469872.1.5.643.
5. Sukul, P.; Spiteller, M. Fluoroquinolone Antibiotics in the Environment. *Rev Environ Contam Toxicol* **2007**, *191*, 131–162, doi:10.1007/978-0-387-69163-3_5.
6. *Reviews of Environmental Contamination and Toxicology 191*; Ware, G., Ed.; Reviews of Environmental Contamination and Toxicology; Springer-Verlag: New York, 2007; ISBN 978-0-387-69162-6.
7. Limberakis, C. Quinolone Antibiotics: Levofloxacin (Levaquin®), Moxifloxacin (Avelox®), Gemifloxacin (Factive®), and Garenoxacin (T-3811). In *The Art of Drug Synthesis*; John Wiley & Sons, Ltd, 2007; pp. 39–69 ISBN 978-0-470-13497-9.
8. Saravolatz, L.D.; Leggett, J. Gatifloxacin, Gemifloxacin, and Moxifloxacin: The Role of 3 Newer Fluoroquinolones. *Clin Infect Dis* **2003**, *37*, 1210–1215, doi:10.1086/378809.
9. Caeiro, J.-P.; Iannini, P.B. Moxifloxacin (Avelox®): A Novel Fluoroquinolone with a Broad Spectrum of Activity. *Expert Review of Anti-infective Therapy* **2003**, *1*, 363–370, doi:10.1586/14787210.1.3.363.
10. Al Omari, MMH; Jaafari, D.S.; Al-Sou'od, K.A.; Badwan, A.A. Chapter Seven - Moxifloxacin Hydrochloride. In *Profiles of Drug Substances, Excipients and Related Methodology*; Brittain, H.G., Ed.; Academic Press, 2014; Vol. 39, pp. 299–431.
11. Avelox, Moxifloxacin Systemic (Moxifloxacin) Dosing, Indications, Interactions, Adverse Effects, and More Available online: https://reference.medscape.com/drug/avelox-moxifloxacin-systemic-moxifloxacin-342537 (accessed on 15 July 2021).
12. Pham, T.D.M.; Ziora, Z.M.; Blaskovich, M.A.T. Quinolone Antibiotics. *Medchemcomm* **2019**, *10*, 1719–1739, doi:10.1039/c9md00120d.
13. Anonymous Factive: Withdrawn Application Available online: https://www.ema.europa.eu/en/medicines/human/withdrawn-applications/factive (accessed on 14 July 2021).
14. Factive (Gemifloxacin) Dosing, Indications, Interactions, Adverse Effects, and More Available online: https://reference.medscape.com/drug/factive-gemifloxacin-342529 (accessed on 15 July 2021).
15. Tótoli, E.G.; Salgado, H.R.N. Besifloxacin: A Critical Review of Its Characteristics, Properties, and Analytical Methods. *Critical Reviews in Analytical Chemistry* **2018**, *48*, 132–142, doi:10.1080/10408347.2018.1429885.
16. Besifloxacin Ophthalmic (Rx) (Voreloxin) Dosing, Indications, Interactions, Adverse Effects, and More Available online: https://reference.medscape.com/drug/besivance-besifloxacin-ophthalmic-999210 (accessed on 15 July 2021).
17. Wetzel, C.; Lonneman, M.; Wu, C. Polypharmacological Drug Actions of Recently FDA Approved Antibiotics. *European Journal of Medicinal Chemistry* **2021**, *209*, 112931, doi:10.1016/j.ejmech.2020.112931.
18. XTORO (Finafloxacin Otic Suspension) 0.3% For Topical Otic Administration HIGHLIGHTS OF PRESCRIBING INFORMATION (206307s000lbl.Pdf).
19. Markham, A. Delafloxacin: First Global Approval. *Drugs* **2017**, *77*, 1481–1486, doi:10.1007/s40265-017-0790-5.

20. Baxdela (Delafloxacin) Dosing, Indications, Interactions, Adverse Effects, and More Available online: https://reference.medscape.com/drug/baxdela-delafloxacin-1000153 (accessed on 15 July 2021).
21. HIGHLIGHTS OF PRESCRIBING INFORMATION Besivance™ (Besifloxacin Ophthalmic Suspension) 0.6%.
22. HIGHLIGHTS OF PRESCRIBING INFORMATION CIPRO® (Ciprofloxacin Hydrochloride) Tablet, for Oral Use CIPRO® (Ciprofloxacin Hydrochloride), for Oral Suspension.
23. HIGHLIGHTS OF PRESCRIBING INFORMATION BAXDELA (Delafloxacin) Tablets, for Oral Use BAXDELA (Delafloxacin) for Injection, for Intravenous Use.
24. Cross-Discipline Team Leader Review XTORO (Finafloxacin Otic Suspension) 0.3%.
25. PRESCRIBING INFORMATION FACTIVE®(Gemifloxacin Mesylate) Tablets.
26. HIGHLIGHTS OF PRESCRIBING INFORMATION LEVAQUIN® (Levofloxacin) Tablet, Film Coated for Oral Use LEVAQUIN® (Levofloxacin) Solution for Oral Use LEVAQUIN® (Levofloxacin) Injection, Solution, Concentrate for Intravenous Use LEVAQUIN® (Levofloxacin) Injection, Solution for Intravenous Use.
27. HIGHLIGHTS OF PRESCRIBING INFORMATION AVELOX (Moxifloxacin Hydrochloride) Tablets, for Oral Use AVELOX (Moxifloxacin Hydrochloride) Injection, for Intravenous Use.
28. Core Safety Profile Active Substance: Nadifloxacin Pharmaceutical Form(s)/Strength: 1% Cream.
29. NegGram® Caplets (Nalidixic Acid, USP).
30. TABLETS NOROXIN® (NORFLOXACIN).
31. FLOXIN® Tablets (Ofloxacin Tablets).
32. Pefloxacin Available online: https://go.drugbank.com/drugs/DB00487 (accessed on 21 October 2021).
33. WHO Publishes List of Bacteria for Which New Antibiotics Are Urgently Needed Available online: https://www.who.int/news/item/27-02-2017-who-publishes-list-of-bacteria-for-which-new-antibiotics-are-urgently-needed (accessed on 3 July 2021).
34. TYGACIL® (Tigecycline) FOR INJECTION for Intravenous Use - PRESCRIBING INFORMATION (021821s021lbl.Pdf).
35. Wang, L.; Liu, D.; Lv, Y.; Cui, L.; Li, Y.; Li, T.; Song, H.; Hao, Y.; Shen, J.; Wang, Y.; et al. Novel Plasmid-Mediated Tet(X5) Gene Conferring Resistance to Tigecycline, Eravacycline, and Omadacycline in a Clinical Acinetobacter Baumannii Isolate. *Antimicrob Agents Chemother* **2019**, *64*, e01326-19, doi:10.1128/AAC.01326-19.
36. ZERBAXA® (Ceftolozane and Tazobactam) for Injection, for Intravenous Use - PRESCRIBING INFORMATION (Zerbaxa_pi.Pdf).
37. Wi, YM; Greenwood-Quaintance, K.E.; Schuetz, A.N.; Ko, KS; Peck, K.R.; Song, J.-H.; Patel, R. Activity of Ceftolozane-Tazobactam against Carbapenem-Resistant, Non-Carbapenemase-Producing Pseudomonas Aeruginosa and Associated Resistance Mechanisms. *Antimicrob Agents Chemother* **2017**, *62*, e01970-17, doi:10.1128/AAC.01970-17.
38. AVYCAZ (Ceftazidime and Avibactam) for Injection, for Intravenous Use - PRESCRIBING INFORMATION (Avycaz_Final_PI_CBE-0_10_2019.Pdf).
39. Wang, Y.; Wang, J.; Wang, R.; Cai, Y. Resistance to Ceftazidime–Avibactam and Underlying Mechanisms. *Journal of Global Antimicrobial Resistance* **2020**, *22*, 18–27, doi:10.1016/j.jgar.2019.12.009.
40. Wise, MG; Estabrook, M.A.; Sahm, D.F.; Stone, G.G.; Kazmierczak, K.M. Prevalence of Mcr-Type Genes among Colistin-Resistant Enterobacteriaceae Collected in 2014-2016 as Part of the INFORM Global Surveillance Program. *PLoS One* **2018**, *13*, e0195281, doi:10.1371/journal.pone.0195281.
41. de Jonge, BLM; Karlowsky, J.A.; Kazmierczak, K.M.; Biedenbach, D.J.; Sahm, D.F.; Nichols, W.W. In Vitro Susceptibility to Ceftazidime-Avibactam of Carbapenem-Nonsusceptible Enterobacteriaceae Isolates Collected

during the INFORM Global Surveillance Study (2012 to 2014). *Antimicrob Agents Chemother* **2016**, *60*, 3163–3169, doi:10.1128/AAC.03042-15.

42. Kazmierczak, K.M.; Bradford, P.A.; Stone, G.G.; de Jonge, B.L.M.; Sahm, D.F. In Vitro Activity of Ceftazidime-Avibactam and Aztreonam-Avibactam against OXA-48-Carrying Enterobacteriaceae Isolated as Part of the International Network for Optimal Resistance Monitoring (INFORM) Global Surveillance Program from 2012 to 2015. *Antimicrob Agents Chemother* **2018**, *62*, e00592-18, doi:10.1128/AAC.00592-18.
43. Kazmierczak, K.M.; de Jonge, B.L.M.; Stone, G.G.; Sahm, D.F. In Vitro Activity of Ceftazidime/Avibactam against Isolates of Enterobacteriaceae Collected in European Countries: INFORM Global Surveillance 2012-15. *J Antimicrob Chemother* **2018**, *73*, 2782–2788, doi:10.1093/jac/dky266.
44. Sader, H.S.; Castanheira, M.; Shortridge, D.; Mendes, R.E.; Flamm, R.K. Antimicrobial Activity of Ceftazidime-Avibactam Tested against Multidrug-Resistant Enterobacteriaceae and Pseudomonas Aeruginosa Isolates from US Medical Centers, 2013 to 2016. *Antimicrob Agents Chemother* **2017**, *61*, e01045-17, doi:10.1128/AAC.01045-17.
45. Sader, H.S.; Castanheira, M.; Mendes, R.E.; Flamm, R.K. Frequency and Antimicrobial Susceptibility of Gram-Negative Bacteria Isolated from Patients with Pneumonia Hospitalised in ICUs of US Medical Centres (2015-17). *J Antimicrob Chemother* **2018**, *73*, 3053–3059, doi:10.1093/jac/dky279.
46. Senchyna, F.; Gaur, RL; Sandlund, J.; Truong, C.; Tremintin, G.; Kültz, D.; Gomez, C.A.; Tamburini, F.B.; Andermann, T.; Bhatt, A.; et al. Diversity of Resistance Mechanisms in Carbapenem-Resistant Enterobacteriaceae at a Health Care System in Northern California, from 2013 to 2016. *Diagn Microbiol Infect Dis* **2019**, *93*, 250–257, doi:10.1016/j.diagmicrobio.2018.10.004.
47. Yin, D.; Wu, S.; Yang, Y.; Shi, Q.; Dong, D.; Zhu, D.; Hu, F.; China Antimicrobial Surveillance Network (CHINET) Study Group Results from the China Antimicrobial Surveillance Network (CHINET) in 2017 of the In Vitro Activities of Ceftazidime-Avibactam and Ceftolozane-Tazobactam against Clinical Isolates of Enterobacteriaceae and Pseudomonas Aeruginosa. *Antimicrob Agents Chemother* **2019**, *63*, e02431-18, doi:10.1128/AAC.02431-18.
48. Karlowsky, J.A.; Kazmierczak, K.M.; Bouchillon, S.K.; de Jonge, B.L.M.; Stone, G.G.; Sahm, D.F. In Vitro Activity of Ceftazidime-Avibactam against Clinical Isolates of Enterobacteriaceae and Pseudomonas Aeruginosa Collected in Asia-Pacific Countries: Results from the INFORM Global Surveillance Program, 2012 to 2015. *Antimicrob Agents Chemother* **2018**, *62*, e02569-17, doi:10.1128/AAC.02569-17.
49. Hackel, M.; Kazmierczak, K.M.; Hoban, D.J.; Biedenbach, D.J.; Bouchillon, S.K.; de Jonge, BLM; Stone, G.G. Assessment of the In Vitro Activity of Ceftazidime-Avibactam against Multidrug-Resistant Klebsiella Spp. Collected in the INFORM Global Surveillance Study, 2012 to 2014. *Antimicrob Agents Chemother* **2016**, *60*, 4677–4683, doi:10.1128/AAC.02841-15.
50. Flamm, R.K.; Nichols, W.W.; Sader, H.S.; Farrell, D.J.; Jones, R.N. In Vitro Activity of Ceftazidime/Avibactam against Gram-Negative Pathogens Isolated from Pneumonia in Hospitalised Patients, Including Ventilated Patients. *Int J Antimicrob Agents* **2016**, *47*, 235–242, doi:10.1016/j.ijantimicag.2016.01.004.
51. Wilson, W.R.; Kline, E.G.; Jones, C.E.; Morder, K.T.; Mettus, R.T.; Doi, Y.; Nguyen, M.H.; Clancy, C.J.; Shields, R.K. Effects of KPC Variant and Porin Genotype on the In Vitro Activity of Meropenem-Vaborbactam against Carbapenem-Resistant Enterobacteriaceae. *Antimicrob Agents Chemother* **2019**, *63*, e02048-18, doi:10.1128/AAC.02048-18.
52. Gonzalez, M.D.; McMullen, A.R.; Wallace, M.A.; Crotty, M.P.; Ritchie, D.J.; Burnham, C.A.D. Susceptibility of Ceftolozane-Tazobactam and Ceftazidime-Avibactam Against a Collection of β-Lactam-Resistant Gram-Negative Bacteria. *Ann Lab Med* **2017**, *37*, 174–176, doi:10.3343/alm.2017.37.2.174.

53. Kazmierczak, K.M.; de Jonge, B.L.M.; Stone, G.G.; Sahm, D.F. In Vitro Activity of Ceftazidime/Avibactam against Isolates of Pseudomonas Aeruginosa Collected in European Countries: INFORM Global Surveillance 2012-15. *J Antimicrob Chemother* **2018**, *73*, 2777–2781, doi:10.1093/jac/dky267.
54. Sader, H.S.; Castanheira, M.; Flamm, R.K.; Mendes, R.E.; Farrell, D.J.; Jones, R.N. Ceftazidime/Avibactam Tested against Gram-Negative Bacteria from Intensive Care Unit (ICU) and Non-ICU Patients, Including Those with Ventilator-Associated Pneumonia. *Int J Antimicrob Agents* **2015**, *46*, 53–59, doi:10.1016/j.ijantimicag.2015.02.022.
55. Hachem, R.; Reitzel, R.; Rolston, K.; Chaftari, A.-M.; Raad, I. Antimicrobial Activities of Ceftazidime-Avibactam and Comparator Agents against Clinical Bacteria Isolated from Patients with Cancer. *Antimicrob Agents Chemother* **2017**, *61*, e02106-16, doi:10.1128/AAC.02106-16.
56. FETROJA (Cefiderocol) for Injection, for Intravenous Use - PRESCRIBING INFORMATION (Fetroja.Pdf).
57. Malik, S.; Kaminski, M.; Landman, D.; Quale, J. Cefiderocol Resistance in Acinetobacter Baumannii: Roles of β-Lactamases, Siderophore Receptors, and Penicillin Binding Protein 3. *Antimicrob Agents Chemother* **2020**, *64*, e01221-20, doi:10.1128/AAC.01221-20.
